# Targeting PRAME directly or via EZH2 inhibition overcomes retinoid resistance and represents a novel therapy for keratinocyte carcinoma

**DOI:** 10.1002/1878-0261.13820

**Published:** 2025-03-18

**Authors:** Brandon Ramchatesingh, Amelia Martinez Villarreal, Philippe Lefrançois, Jennifer Gantchev, Sriraam Sivachandran, Samy Abou Setah, Ivan V. Litvinov

**Affiliations:** ^1^ Division of Experimental Medicine McGill University Montreal Canada; ^2^ Faculty of Medicine and Health Sciences McGill University Montreal Canada; ^3^ Division of Dermatology McGill University Health Center Montreal Canada; ^4^ Lady Davis Institute for Medical Research, Jewish General Hospital McGill University Montreal Canada

**Keywords:** basal cell carcinoma, cutaneous squamous cell carcinoma, EZH2, PRAME, retinoid resistance, retinoids

## Abstract

Retinoids have demonstrated efficacy as preventative/treatment agents for keratinocyte carcinomas (KCs): basal cell carcinoma (BCC) and cutaneous squamous cell carcinoma (SCC). However, retinoid resistance mechanisms limit the efficacy of these compounds. A subset of KCs expresses Preferentially Expressed Antigen in Melanoma (PRAME): a retinoid signaling corepressor. PRAME is proposed to repress retinoid signaling by guiding enhancer of zeste homolog 2 (EZH2) to retinoic acid response elements (RARE) in promoters. We investigated the effects of PRAME on KC pathogenesis and retinoid response. High‐PRAME expression in tumors was negatively correlated with epidermal differentiation gene signatures. PRAME overexpression downregulated epidermal differentiation gene signatures and impaired differentiation in 3D culture. PRAME overexpression attenuated retinoid‐induced RARE activation, growth suppression, and differentiation responses. Conversely, low‐PRAME tumors and PRAME‐depleted KC cells demonstrated enriched epidermal differentiation gene signatures. PRAME downregulation restored retinoid‐induced RARE activation, growth suppression, keratinization in SCC, and cell death signaling in BCC. Furthermore, combined retinoid and EZH2 inhibitor treatment augmented RARE activation and suppressed PRAME‐expressing KC cell growth. Hence, PRAME confers retinoid resistance in KC, which may be overcome by EZH2 inhibition.

## Graphical abstract

The study evaluated the function and therapeutic implications of PRAME in basal cell carcinoma (BCC) and squamous cell carcinoma (SCC). The findings demonstrate that PRAME impairs keratinocyte differentiation pathways. Furthermore, PRAME impairs anticancer response to retinoid compounds in BCC and SCC cells. Anticancer response to retinoid treatment was restored by combining retinoid treatment with use of an EZH2 inhibitor.

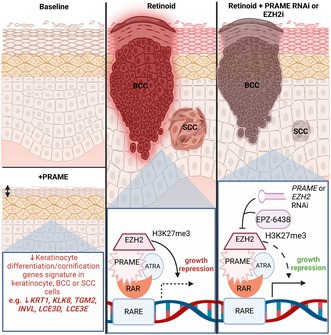

**Abbreviations**
AK: actinic keratosis
ANOVA: analysis of variance
ATRA: all‐trans retinoic acid
BCC: basal cell carcinoma
cSCC: cutaneous squamous cell carcinoma
cvSCC: cervical squamous cell carcinoma
DEG: differentially expressed gene
DMSO: dimethyl sulfoxide
EZH2: enhancer of zeste homolog 2
GSEA: gene set enrichment analysis
HNSCC: head and neck squamous cell carcinoma
KC: keratinocyte carcinoma
PRAME: preferentially expressed antigen in melanoma
RAR: retinoic acid receptor
RARE: retinoic acid response element
RXR: retinoid X receptor
SCC: squamous cell carcinoma
SEM: standard error of the mean
TCGA: The Cancer Genome Atlas

## 1 Introduction

Keratinocyte carcinomas (KC) are nonmelanoma skin cancers (NMSCs) that arise from epidermal keratinocytes [1, 2, 3, 4]. The two types of KC are basal cell (BCC) and cutaneous squamous cell carcinomas (cSCC). BCC and cSCC represent the first and the fifth most common malignancies worldwide, respectively [5, 6, 7]. The estimated incidence of KC is 407 cases per 100 000 Caucasian men and 212 cases per 100 000 Caucasian women in the United States, and as high as > 800 cases per 100 000 individuals in Australia [5, 6]. Troubling increases in KC incidence have been documented in recent decades [5, 6, 8]. KC can be locally destructive, have mutilating effects, and cause psychological distress in patients [9, 10]. Surgical and ablative therapies, the standard of care for localized disease, are invasive and can be disfiguring [10].

BCC and cSCC arise from distinct subpopulations of interfollicular epidermal keratinocytes and hair follicle stem cells [2, 3, 4, 11, 12, 13]. In the healthy epidermis, basal layer keratinocytes proceed through a multi‐step terminal differentiation process, ending with the formation of the cornified envelope/cornification [14]. Cornification is the process whereby keratinocytes form the uppermost differentiated layer of the epidermis, the stratum corneum [14]. Terminally differentiated keratinocytes express hallmark keratin proteins (e.g., keratin 1/10), crosslinking proteins (e.g., transglutaminases), and cornified envelope proteins (e.g., involucrin) [14]. Aberrant expression or lack of terminal differentiation markers in cancer cells suggests the induction of phenotypic plasticity, manifesting as poorly differentiated, aggressive disease [15, 16]. Transcriptional signatures related to keratinization and cornification are downregulated in BCC when compared to healthy control tissues [17]. Similarly, SCCs display transcriptional signatures indicating aberrant upregulation of squamous differentiation and keratinization pathways [18, 19]. The histological hallmark of SCC are keratin pearls which are eosinophilic spirals of squamous cells overproducing keratin proteins [19].

Retinoids are the natural and synthetic derivatives of retinol (vitamin A) [20]. Retinoids function as agonists for retinoic acid receptors (RAR) and retinoid × receptors (RXR). According to the canonical retinoid signaling pathway (excluding noncanonical and alternate receptor pathways) [21], retinoids undergo import into cells and enzymatic conversion to bioactive retinoid ligands, such as all‐trans retinoic acid (ATRA). Bioactive retinoid ligands bind to RAR‐RXR heterodimers positioned at retinoic acid response elements (RARE) within gene promoters [22]. Ligand binding initiates exchange of a transcriptional corepressor complex for a transcriptional coactivator complex that opens chromatin at the RARE and enables recruitment of transcriptional machinery to the promoter [22]. Conversely, RAR‐RXR heterodimers may also recruit corepressor complexes upon ligand binding [23]. Retinoids exert transcriptional control over genes that govern cell differentiation, growth arrest, and cell death programs [22, 24]. Provided their effects upon these processes, retinoids have been studied and applied as therapeutic and preventative agents for multiple cancers [22].

Retinoids are potent regulators of skin physiology with demonstrated efficacy in KC prevention and treatment (especially for treatment of *in situ* disease or precancerous actinic keratoses). To date, four generations of topical and systemic retinoids have been developed and used to manage skin disorders such as acne, psoriasis, disorders of keratinization, cutaneous lymphomas, and Kaposi Sarcomas [25, 26]. In the epidermis, retinoids normalize keratinocyte turnover: the balance between proliferation, differentiation, and cell death [21]. Extending their activity to malignant contexts, retinoids initiate antineoplastic processes in BCC and cSCC cells. In BCC cells, retinoids promote cell death and control the oncogenic Shh‐PTCH1‐Gli and PI3K‐Akt–mTOR pathways [27, 28, 29]. In cSCC cells, retinoids regulate oncogenic STAT3, MAPK pathway activation, and cooperate with the interferon signaling axis to suppress cell proliferation [30, 31]. Retinoids also normalize overactive squamous differentiation programs in cSCC cells, restraining excess keratinization [32, 33, 34]. Although the mechanisms underlying this control are unclear, retinoids have been shown to control expression of vital regulators of keratinization and squamous differentiation (e.g., NOTCH1) [18, 35, 36].

Systemic retinoids (e.g., oral acitretin) are recommended for KC chemoprevention in predisposed individuals [37, 38]. Topical retinoids are routinely used to prevent and treat AKs [21, 39]. Numerous reports have documented successful use of retinoid therapeutics to regress KC tumors [21]. Nonetheless, clinical trials have reported subsets of patients who respond poorly or who were unresponsive to retinoids, preventing the incorporation of retinoids into KC treatment guidelines [21, 40, 41, 42]. Suboptimal retinoid responses may be attributable to retinoid resistance [24]. Retinoid resistance in cancer occurs due to disruptions in retinoid signaling pathways [22, 43]. Understanding and addressing the molecular determinants of retinoid resistance is essential for optimizing the use of these drugs to manage KC.

Preferentially expressed antigen in melanoma (PRAME) is a cancer‐testis antigen that acts as a transcriptional repressor of retinoid signaling [44]. PRAME is a leucine‐rich repeat protein that is expressed exclusively in gametogenic cells in healthy individuals [45, 46]. In gametes, PRAME and its family members regulate cell growth, differentiation, and retinoid signaling [47, 48, 49]. Ectopic PRAME expression has been reported in several types of cancer, where it has been associated with poor prognoses and aggressive disease, including invasive disease and accelerated tumor growth [50, 51, 52, 53, 54, 55]. PRAME exerts its retinoid repressor function by binding to RARs and binding to enhancer of zeste homolog 2 (EZH2), a histone methyltransferase. EZH2 deposits the repressive histone modification H3K27me3. By binding to RARs and to EZH2, PRAME guides H3K27me3 deposition to RARE elements, thereby attenuating retinoid‐induced transcription [44]. Additional roles for PRAME in cancer have been described. For example, PRAME associates with Cullin 2 RING E3 ubiquitin ligases [56], thereby mediating the degradation of important cell signaling regulators, including p14/ARF [57, 58].

PRAME is ectopically expressed in subsets of BCC (> 60%) and cSCC (~ 40%) tumors [59, 60, 61, 62]. Moderate‐ to poorly differentiated cSCC, representing aggressive disease, exhibit higher PRAME expression compared to well‐differentiated cSCC [60]. The clinical relevance of PRAME expression in KC and its impact upon KC biology and treatment responses have not been studied. This study investigates the effects of PRAME expression on cell phenotypes and response to retinoid‐based therapy in KC.

## 2 Materials and methods

### 2.1 Cell culture and tissue collection

All cell lines are human. HaCaT cells (RRID:CVCL_0038) were provided by the laboratory of Dr. A. Philip (Montreal General Hospital, QC, CA). Ker‐CT (ATCC #CRL‐4048; RRID:CVCL_S877), A431 (ATCC #CRL‐1555; RRID:CVCL_0037), CAL‐27 (#CRL‐2095; RRID:CVCL_1107), and UPCI:SCC154 (ATCC, #CRL‐3241; RRID:CVCL_2230) cell lines were purchased from ATCC. Primary human neonatal dermal fibroblasts (HDF, Cell Applications Inc., #106‐05n, San Diego, CA, USA) were purchased from Cell Applications Inc. UW‐BCC1 cells were provided by the laboratory of Dr. V. Spiegelman (Pennsylvania State University, PA, USA) [63]. Ker‐CT cells were cultured in Keratinocyte Basal Medium Gold® (KBM‐Gold) with bullet kit supplements (Lonza, #00192060, Durham, NC, USA). HaCaT, A431, CAL‐27, UW‐BCC1, and HDF were cultured in Dulbecco's modified Eagle medium (DMEM, ATCC #30‐2002) supplemented with 10% heat‐inactivated fetal bovine serum (FBS, Thermo Fisher Scientific, #12484028, Waltham MA, USA) and 1% penicillin streptomycin (Thermo Fisher Scientific, #15140122). UPCI: SCC‐154 was cultured in Eagle's Minimum Essential Medium (EMEM, ATCC, #30‐2003) supplemented with 10% FBS, 1% penicillin streptomycin and 1% L‐glutamine (Thermo Fisher Scientific, #25030081). Cells were maintained in a humidified 37 °C incubator with 5% CO_2_.

Cell line authentication was performed by ATCC within the past 3 years using Short Tandem Repeat profiling, as a part of their routine authentication and quality control testing of cell line distribution lots. Cells were maintained for a maximum of 25 passages. Cells were routinely tested for mycoplasma contamination.

Pathologically confirmed tumor tissues and healthy control tissue samples were flash frozen and stored in liquid nitrogen. All experiments on human tumor samples were undertaken with the understanding and written informed consent of the tissue donors. This study was approved by the Institutional Review Board at the Research Institute of the McGill University Health Center (IRB protocols #2018‐4128 and #2022‐8414). Study subject age, sex, and pathological diagnosis are provided in Table S1. The study methodology conformed to the standards set by the Declaration of Helsinki.

### 2.2 Drug treatments

All‐trans retinoic acid (ATRA, Sigma‐Aldrich, #R2625‐50MG, St Louis, MS, USA), tazarotene (Sigma‐Aldrich, #T7080), and tazemetostat (EPZ‐6438, Selleckchem, #S7128, Houston, TX, USA) were dissolved in dimethyl sulfoxide (DMSO). All retinoid treatments were performed in the dark, and treated culture vessels were protected from light. Drug concentrations were optimized and chosen based on previous reports and by conducting proliferation assays and evaluating response markers by western blotting.

### 2.3 Lentiviral transduction

All prepackaged and predesigned lentiviral transduction products were purchased from Dharmacon Revvity (Waltham, MA, USA). Product specifications are provided in Tables S2–S4. Cells were plated at an optimized density and transduced with a lentivirus multiplicity of infection (MOI) optimized according to each cell line. Transductions were performed in the presence of 8 μg·mL^−1^ polybrene transduction reagent (Millipore Sigma, #TR‐1003‐G, Oakville, Ontario, Canada). Transductions using *PRAME* Precision Lenti‐ORF lentivirus and empty vector (EV) lentiviral particles were performed to generate *PRAME* overexpressing (*PRAME* OE) and EV control cell lines [64]. Transduced cells were selected for using optimized concentrations of blasticidin. SMARTvector *PRAME* lentiviral shRNA and scrambled shRNA (SCR) transductions were performed to generate *PRAME* knockdown (KD) and SCR control cell lines. Transduced cells were selected for using optimized concentrations of puromycin. Monoclonal populations were raised from single cells. Edit‐R All‐in‐one lentiviral *PRAME* sgRNA and scrambled sgRNA (sgSCR) transductions were performed to generate *PRAME* knockout (KO) and sgSCR control cell lines. Transduced cells were selected for using puromycin. Monoclonal populations were raised from single cells. Successful *PRAME* OE, KD, and KO were evaluated by western blotting and RT‐qPCR.

### 2.4 Protein isolation, western blotting, RNA isolation, and RT‐qPCR

Protein isolation [65], western blotting [66], RNA isolation [67, 68, 69], and RT‐qPCR [70, 71, 72, 73] were performed as previously described. Antibody sources and dilutions are provided in Table S5. Blots were developed in a Bio‐Rad ChemiDoc™ Touch Imaging system (Bio‐Rad, #12003153, Mississauga, Ontario, Canada) using Clarity™ Western ECL Substrate (Bio‐Rad, #1705061). Images were visualized using imagelab software (v6.0.1, RRID: SCR_014210, Bio‐Rad). Full uncropped blots are provided in Fig. S7. The following primers were used (5′ to 3′): *PRAME* Fwd: CTCAGCACCGCTCCGGGACA, *PRAME* Rev: CACCCCGCCCCGCAAGTCTA, *B2M* Fwd: TGC TGT CTC CAT GTT TGA TGT ATC T, *B2M* Rev: TCT CTG CTC CCC ACC TCT AAG T. *B2M* was used as a housekeeping gene. Relative gene expression was compared using the ΔΔ*C*_T_ method [74].

### 2.5 RNA sequencing and data processing

RNA for sequencing was isolated using the Qiagen RNEasy Mini Kit (Qiagen, #74104, Germantown, MD, USA), as per the manufacturer's protocol. RNA concentration and purity were determined using the BioDrop μLITE (MBI, #80‐3006‐55) spectrophotometer. Genome Québec performed RNA quality control validation and sequencing. RNA was quantified and integrity was assessed using 5K/RNA/Charge Variant Assay LabChip (Perkin Elmer, Waltham MA, USA) and RNA Assay Reagent Kit on a LabChip GXII. Libraries were generated using 250 ng of total RNA. Illumina Stranded mRNA Library Prep kit (Illumina, San Diego, CA, USA) was used per the manufacturer's recommendation for mRNA enrichment and library preparation. Libraries were quantified with KAPA library quantification kits‐Complete Kit (universal) (KAPA Biosystems, Wilmington, MA, USA). Agilent fragment analyzer was used to determine average fragment size (Agilent Technologies, Santa Clara, CA, USA). Libraries were normalized, pooled, and denatured with 0.2 m NaOH. HT1 buffer was used for neutralization. The pool was loaded at 175pM onto an Illumina NovaSeq S4 lane with Xp protocol as per the manufacturer's recommendations. The run was performed at 2 × 100 cycles (paired‐end mode). A phiX library was used as a control and mixed with libraries at 1% level. Base calling was performed using rta v3, Illumina. FASTQ reads were demultiplexed and generated using bcl2fastq2 v.2.20. The RNomics platform at the Université de Sherbrooke performed RNA‐Seq analyses. FASTQ files reads were trimmed using trimmomatic (V0.36) [75]. Read quality was evaluated using the fastqc tool (V0.11.5) [76]. kallisto (V0.48.0) [77] was used to pseudo‐align the reads to the GRCh38 transcriptome generated from Ensembl annotation and genome files (V110) [78] using the gffread tool (cufflinks V2.2.1) [79]. The tximport package (V1.22.0) [80] summarized kallisto count estimates at the gene level, and DESeq2 (V1.34) was then employed to identify Differentially Expressed Genes (DEGs) between conditions, utilizing the default Benjamini and Hochberg correction method [81].

### 2.6 Analysis of bioinformatic data

Venn diagrams were made with Biovenn (https://www.biovenn.nl/) [82]. Genes that were determined to be differentially expressed by DESeq2 (*P*‐value < 0.05) were inputted and compared. Bulk mRNA sequencing data from head and neck squamous cell carcinoma (HNSCC) and cervical squamous cell carcinoma (cvSCC) tumors from The Cancer Genome Atlas (TCGA, RRID:SCR_003193) project were consulted using CBioPortal (RRID:SCR_014555) [83, 84, 85]. Tumors were binned according to *PRAME* mRNA expression (RNA‐Seq V2 RSEM) relative to all samples. Tumors sorted into z‐score bins equal to and below −1.2 (bottom 10% of PRAME‐expressing tumors) were grouped into low‐*PRAME*‐expressing groups, while tumors sorted into z‐score bins equal to and above 1.2 (top 10% of *PRAME*‐expressing tumors) were grouped into high‐*PRAME*‐expressing groups. DEGs between groups were computed using cBioPortal. Gene set enrichment analyses (GSEA) were performed according to guidelines by Reimand et al. [86]. GSEA for gene lists unaccompanied by expression values (cBioPortal analyses) were performed using gProfiler (RRID:SCR_006809) [87]. GSEA of RNA‐Seq data and generation of enrichment plots were conducted using the gsea desktop application software (v4.3.3 RRID:SCR_003199) [88].

### 2.7 3D‐organotypic skin culture

3D Organotypic skin culture was performed as described by Marinova et al. [89]. Organoid culture media was prepared as previously described, without cholera toxin [89]. After a total of 14 days at the air–liquid interface (ALI), organoids were fixed with 10% neutral buffered formalin for 72 h and subjected to standard IHC protocols. To generate tumoroid models, Ker‐CT keratinocytes were mixed with cancer cell lines at a ratio of 10 : 1 at the time of seeding the fibro‐collagen beds [90]. For treatment studies, ALI culture was performed up to day 7, and then, drugs were added daily to culture media until day 14, at which time organoids were fixed.

### 2.8 H&E staining and immunohistochemistry (IHC)

H&E staining and IHC were performed as previously described [72]. For PRAME IHC, Tris EDTA pH 9.0 was used for antigen retrieval. Anti‐PRAME (Abcam, #Ab219650) at a dilution of 1 : 250 was used for primary antibody incubation. Slides were scanned with the assistance of the RI‐MUHC Histopathology platform and visualized on the Aperio ImageScope system (RRID:SCR_020993). For analysis of tissue thickness, measurements from 10 images of the tissue sections were obtained (per biological replicate, *n* = 3) using imagej [91] (RRID:SCR_003070).

### 2.9 Generation of RAR‐TRE‐mStrawberry reporter cell lines

RAR‐TRE‐mStrawberry reporter plasmid was generated by the lab of Dr. R. Straussman (Addgene plasmid # 158683, Watertown, MA, USA; http://n2t.net/addgene:158683; RRID: Addgene_158 683) [92]. Plasmid‐transformed *E. coli* were purchased from Addgene and expanded as per the distributor's protocol. Plasmid DNA was isolated using the CompactPrep® Plasmid Midi Kit (Qiagen, #12843). Cells were transfected with 1 μg of plasmid DNA per well using the Lipofectamine 3000 kit (Thermo Fisher Scientific, #L3000001). After transfection, cells were maintained under blasticidin selection.

### 2.10 Clonogenic assay

Clonogenic assay was performed as previously described [93]. Cells were seeded into 6‐well plates at a density of 1000 cells per well and permitted to adhere for 24 h prior to the start of drug treatments. For UW‐BCC1 cells, wells were precoated with poly‐L‐lysine (Sigma‐Aldrich, #P4707‐50ML) prior to seeding. Cells were subjected to daily drug treatments. Colonies of at least 50 cells were counted using a light microscope. Whole‐well counts were obtained. The surviving fraction of colonies relative to DMSO‐treated groups were computed as described [93].

### 2.11 IncuCyte® live‐cell proliferation analysis

Cells were seeded into 96‐well plates at a density of 1000 cells per well or 2500 cells per well for A431 cells. For UW‐BCC1 cells, wells were precoated with poly‐L‐lysine (Sigma‐Aldrich, #P4707‐50ML). Cells were permitted to adhere and grow for 24 h prior to starting the protocol. Imaging was performed using the IncuCyte® (Sartorius Canada Inc., Oakville, Ontario, Canada) S3 live‐cell analysis system (Sartorius, Gottingen, Germany, RRID:SCR_023147). Whole‐well scans were acquired every 6 h over a period of 24–120 h according to the experiment. incucyte® 2021 software was used to apply a confluence‐based analysis definition to plot cell growth (as a function of well confluence) over time.

### 2.12 Immunofluorescence staining

Cells were plated in 6‐well plates at a density of 300 000 cells per well onto sterile glass coverslips, and treatments were performed 24 h after plating. Immunofluorescence staining was performed as previously described 24 h after treatment [72, 94]. 100 μL of anti‐Ki‐67 (Thermo Fisher, #PA5‐16785, RRID:AB_11000602) antibody diluted to 1 : 400 in 1% BSA was used for primary antibody incubation. Anti‐rabbit IgG Alexa fluor 594 or 488 (Cell Signaling, #8889, Danvers, MA, USA; RRID:AB_2716249 or #4408 RRID:AB_10694704 dilution 1 : 1000) were used for secondary antibody incubations. Cells were mounted on VWR Premium Superfrost™ Plus Micro Slides (VWR, #CA48311‐703) using Fluoroshield® mounting media with DAPI (Sigma‐Aldrich, #F6057). Images were captured at 20× magnification using an Etaluma Lumascope LS720 Microscope (Etaluma, Carlsbad, CA, USA). Imaging parameters were set using controls to gate, and imaging was automated to acquire DAPI, red‐channel and merged images over a 7 × 7 region of interest. 500 cells were counted per condition using qupath (version 0.4.4, RRID:SCR_018257) [95].

### 2.13 siRNA transfection

*EZH2* siRNA clones were purchased from Dharmacon. Transfection was performed using DharmaFECT 1 Transfection reagent (Dharmacon, #T‐2001‐01), according to the manufacturer's protocol. Two siRNA sequence clones were purchased: J‐004218‐06‐0010 (sequence: GAGGACGGCUUCCCAAUAA) and J‐004218‐09‐0010 (sequence: GCAAAUUCUCGGUCUCAAA). Clone J‐004218‐06‐0010 demonstrated strongest efficacy. ON‐TARGET plus nontargeting siRNA #1 (Dharmacon, #D‐001810‐01‐05) was used as a negative control. Knockdown was visualized by western blotting 3 days after transfection.

### 2.14 Statistical analyses and data presentation

Statistical analyses and graph plotting were performed using the prism graphpad 10 software (GraphPad Prism v10.0.3, San Diego, CA, USA, RRID:SCR_002798). At least three independent experiments (with distinct biological replicates) were performed (with each independent experiment containing three technical replicates).

The threshold for statistical significance was *P* < 0.05. Figures were assembled using inkscape (v1.2, RRID:SCR_014479).

## 3 Results

### 3.1 PRAME impairs epidermal differentiation gene signatures in keratinocytes and keratinocyte carcinoma cells

**Fig. 1:**
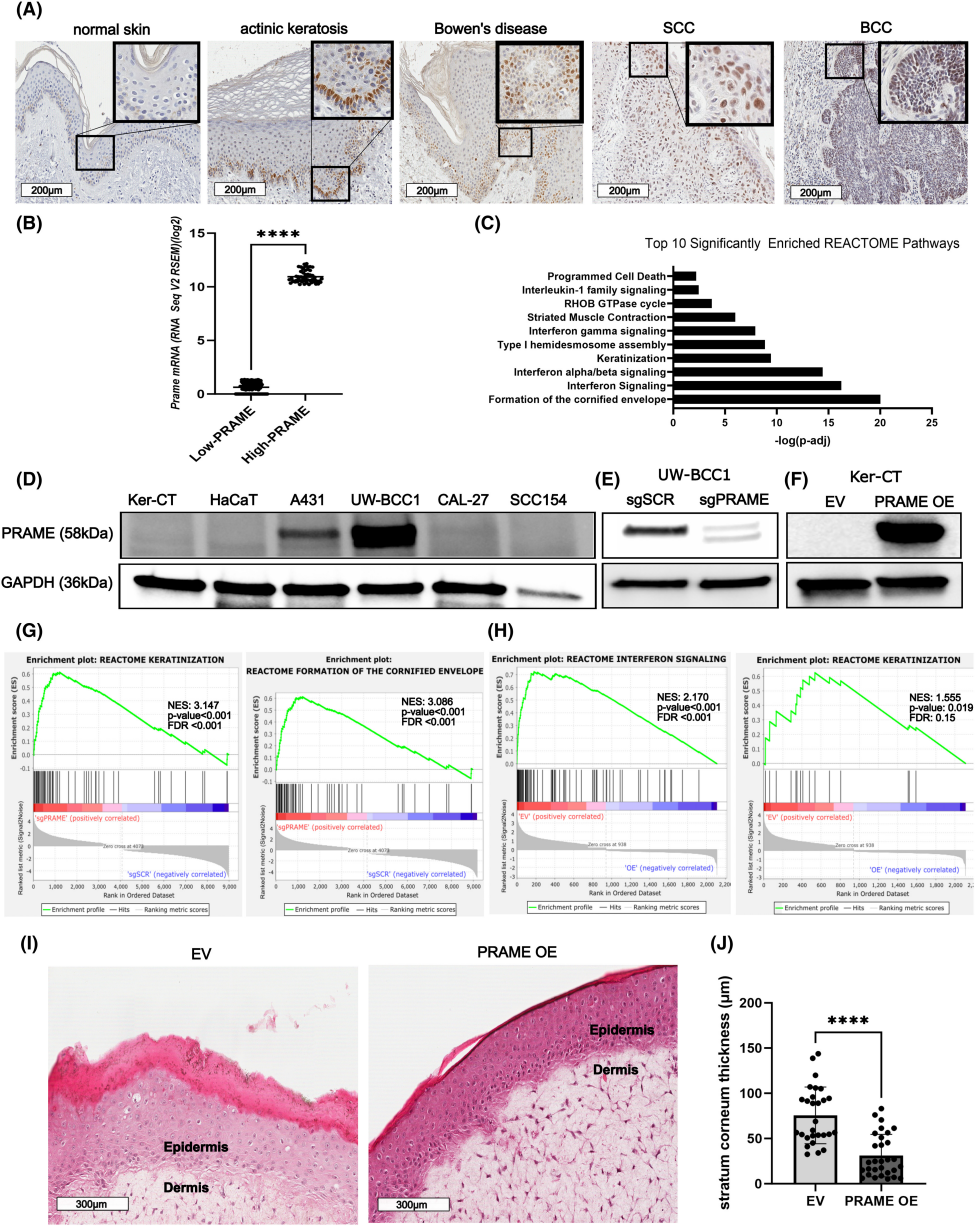
PRAME impairs epidermal differentiation gene signatures in keratinocytes and keratinocyte carcinoma cells. (A) PRAME immunostaining in (from left to right) normal skin, actinic keratosis, Bowen's disease, cSCC, and BCC (Scale bar 200 μm). Insets for high magnification images (40×) visible in the top right corners. (B) *PRAME* mRNA expression (RNA‐seq RSEM V2 log(2)) in the low‐*PRAME* (*n* = 122) and high‐*PRAME* (*n* = 55) expressing Head and Neck SCC (HNSCC) tumors sequenced from TCGA. Statistical significance determined using Student's two‐tailed *t*‐test. (C) REACTOME pathway enrichment for genes significantly upregulated in the low‐*PRAME* group vs. the high‐*PRAME‐*expressing groups. (D) Representative western blot for PRAME in cell lines representative of normal keratinocytes, cSCC, BCC, and HNSCC. GAPDH was probed as a loading control (*n* = 3). (E) Representative western blot for PRAME in scrambled control (sgSCR) and *PRAME* knockout (sg*PRAME*) UW‐BCC1 cells. GAPDH was probed as a loading control (*n* = 3). (F) Representative western blot for PRAME in empty vector (EV) control or *PRAME* overexpression (OE) Ker‐CT cells. GAPDH was probed as a loading control (*n* = 3). (G) Enrichment plots comparing top REACTOME pathways enriched in sg*PRAME* vs. sgSCR UW‐BCC1 cells (*n* = 3). Normalized enrichment score (NES), *P*‐value and false discovery rate (FDR) included. The enrichment plot depicts where genes (black tick marks) associated with a particular biological process are located within the list of DEGs ranked based on correlation with one phenotype versus another. Concentration of tick marks towards one side of the list suggest associations with a given phenotype. Green enrichment profile tracks the computation of the enrichment score (ES). Peak skew towards one side of the graph suggests association of the gene profile with a pathway. For full explanation, see [88]. (H) Enrichment plots comparing top REACTOME pathways enriched in EV vs. *PRAME* OE Ker‐CT cells (*n* = 3). NES, *P*‐value and FDR included. (I) Representative H&E sections of Ker‐CT EV and *PRAME* OE organotypic skin constructs, with epidermal and dermal compartments annotated (scale bar 200 μm) (*n* = 3). (J) Stratum corneum thickness of EV and *PRAME* OE 3D organotypic skin constructs (*n* = 30 measurements, 3 biological replicates/independent experiments, 10 measurements per replicate/independent experiment). Statistical significance determined using Welch's *t*‐test. ****P < 0.0001. Error bars indicate standard error of means (SEM).

We aimed to characterize the expression patterns and biological effects of ectopic PRAME expression in BCC and cSCC. Consistent with several previous reports, in our cohort of skin tumors (Table S1), PRAME protein expression was observed in AKs, Bowen's disease, cSCC and BCC, but not in normal skin (Fig. 1A) [59, 60, 61, 62]. PRAME immunostaining was primarily localized to keratinocyte nuclei, although cytoplasmic staining was observed as well. PRAME expression has been recorded in other keratinocyte‐derived cancer types, including cervical SCC (cvSCC) and head and neck SCC (HNSCC) [96, 97]. To probe relationships between PRAME expression and clinically relevant phenotypes in keratinocyte‐derived malignancies, we analyzed HNSCC and cvSCC mRNA sequencing datasets from TCGA cohorts. Tumors were grouped into low‐ and high‐expressing *PRAME* mRNA groups (Fig. 1B; Fig. S1a), consisting of the bottom 10% and the top 10% of *PRAME* mRNA expressing tumors in the cohort, respectively. Relative to high‐expressing *PRAME* tumors, low‐expressing *PRAME* tumors were enriched in genes associated with the REACTOME pathways formation of the cornified envelope, keratinization, and interferon signaling (Fig. 1C). In cvSCC, pathways such as skin development, keratinocyte differentiation, and keratinization were upregulated in low‐*PRAME* tumors relative to high‐*PRAME* tumors (Fig. S1b). These findings suggest a role for PRAME in modulating keratinocyte differentiation gene signatures.

To investigate whether PRAME actively modifies transcriptional signatures in KC, we first identified suitable patient‐derived keratinocyte and KC cell line models in which to mechanistically study *PRAME* expression. PRAME protein expression was detectable in the A431 cSCC cell line and UW‐BCC1 BCC cell line (Fig. 1D). PRAME protein expression was undetectable in Ker‐CT immortalized keratinocytes and in CAL‐27 and UPCI:SCC‐154 oral SCC cells (Fig. 1D). Therefore, we selected Ker‐CT, CAL‐27, and UPCI:SCC‐154 cells for *PRAME* overexpression (OE) experiments and A431 and UW‐BCC1 for *PRAME* knockdown (KD) and knockout (KO) experiments.

*PRAME* was knocked out in UW‐BCC1 cells and overexpressed in Ker‐CT cells (Fig. 1E,F), and then, bulk RNA‐Seq was performed (Fig. S1c–e). *PRAME* KO cells were enriched for genes associated with the REACTOME pathways keratinization and formation of the cornified envelope (Fig. 1G). Results were recapitulated using GO:BP datasets (Fig. S1f). An increase in late‐cornified envelope genes (e.g., LCE3D) and differentiation‐associated keratins (e.g., KRT1) was noted in sgPRAME cells (Fig. S1g). PRAME KO cells were also enriched in genes associated with interleukin and interferon signaling pathways, relative to control cells (Fig. S1f). Conversely, *PRAME* OE in Ker‐CT cells downregulated the REACTOME pathways and interferon signaling and keratinization (Fig. 1H). Results were recapitulated using GO:BP datasets (Fig. S1h). Keratinization‐associated genes such as *KLK5*, *KLK8*, and *IVL* (Fig. S1i), as well as interferon signaling genes such as *RIG1, OAS1, STAT1*, and *IRF7* were downregulated upon *PRAME* OE (Fig. S1j).

These results led us to hypothesize that PRAME impairs epidermal differentiation processes. Therefore, we created 3D organotypic skin constructs using Ker‐CT EV and *PRAME* OE keratinocytes (Fig. 1I). As a surrogate measure of terminal differentiation, we measured the thickness of the stratum corneum [91]. *PRAME* OE decreased stratum corneum thickness (Fig. 1J). Total skin thickness also decreased in *PRAME* OE organoids (Fig. S1k). These results suggest that PRAME impairs keratinocyte differentiation programs.

### 3.2 PRAME impairs retinoid‐induced growth suppression in KC cell lines

**Fig. 2:**
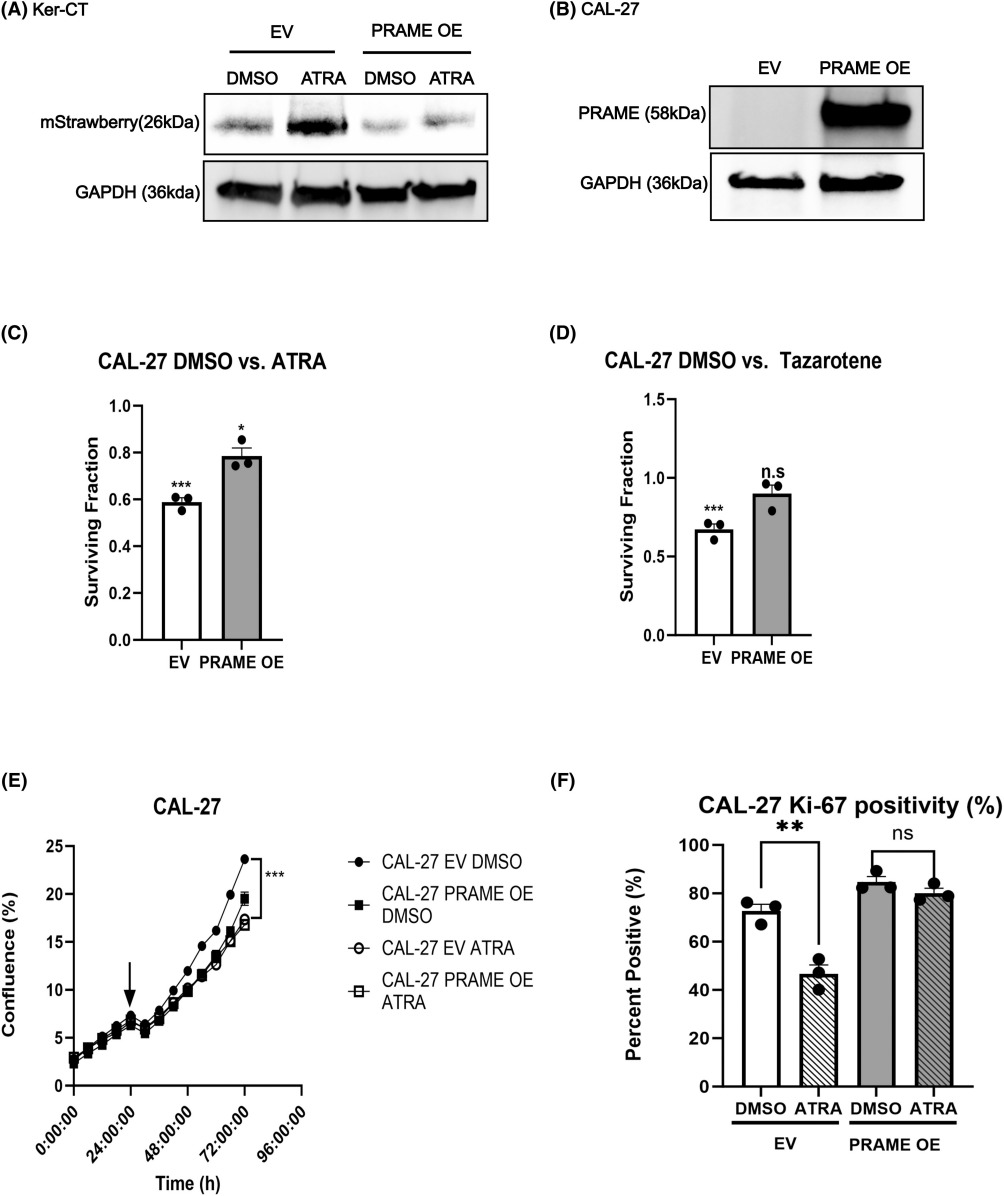
PRAME impairs retinoid‐induced growth suppression in KC cell lines. (A) Western blot for mStrawberry in Ker‐CT EV and *PRAME* OE keratinocytes transfected with the RAR‐TRE‐mStrawberry plasmid and treated with 1 μm ATRA or DMSO. GAPDH was probed as a loading control (*n* = 1). Western blot results were repeated in additional cell lines/clones shown in Fig. S2. (B) Representative western blot for PRAME in EV or *PRAME* OE CAL‐27 cells. GAPDH was probed as a loading control (*n* = 3). (C) Surviving fraction of EV and *PRAME* OE CAL‐27 cells treated with 20 μm ATRA (*n* = 3). DMSO treatment is set at onefold when comparing cell survival in response to drug treatment. Significance was determined by applying Student's two‐tailed *t*‐test to raw colony counts, comparing DMSO control to retinoid treatment conditions. (D) Surviving fraction of EV and PRAME OE CAL‐27 cells treated with 10 μm Tazarotene (*n* = 3). DMSO treatment is set as onefold when comparing cell survival in response to drug treatment. Significance was determined by applying student's two‐tailed *t*‐test to raw colony counts, comparing DMSO control to retinoid treatment conditions [93]. (E) IncuCyte® proliferation analysis of EV and *PRAME* OE CAL‐27 cells treated with 20 μm ATRA or DMSO (*n* = 3). Two‐way ANOVA. (F) Ki‐67 staining index for CAL‐27 EV and *PRAME* OE cells treated with 20 μm ATRA or DMSO (*n* = 3). Student's two‐tailed *t*‐test. ns, not significant, *P < 0.05, **P < 0.01, ***P < 0.001. Error bars indicate SEM.

In the context of KC treatment, the transcriptional output of canonical retinoid signaling is therapeutically relevant [21]. Considering that PRAME can function as a repressor of retinoid‐induced transcription [44], we evaluated whether *PRAME* OE modifies retinoid‐induced transcription in keratinocytes and KC cells. EV and *PRAME* OE Ker‐CT and CAL‐27 cells were subjected to ATRA treatment, followed by bulk RNA‐Seq and transcriptomic analyses. ATRA‐regulated genes were inferred by computing DEGs between DMSO and ATRA‐treated cells. Numerous ATRA‐regulated transcripts are unique to either EV or *PRAME* OE conditions, suggesting that PRAME modifies transcriptional response to retinoid compounds (Fig. S2a,b). PRAME regulates transcription of RARE‐containing genes through associations with RARs [44]. Therefore, we hypothesized that transcription of RARE‐containing genes specifically could be impaired by *PRAME* OE. To evaluate RARE activation, we transfected EV and *PRAME* OE cells with a reporter plasmid containing the mStrawberry protein under the control of a RARE (RAR‐TRE‐mStrawberry) and treated cells with ATRA [92]. Interestingly, increased baseline mStrawberry production was observed in UPCI:SCC154 PRAME OE cells compared to EV cells in the absence of retinoid treatment (Fig. S2c,d). Nonetheless, it was consistently observed that while EV cells exhibit increased mStrawberry protein after ATRA treatment relative to DMSO, PRAME OE cells do not, suggesting that *PRAME* OE impairs transcription of RARE‐containing genes in response to retinoid (Fig. 2A, Fig. S2c,d).

Retinoid responses differ between nontransformed keratinocytes and malignant keratinocytes [21]. Hence, to study the therapeutic implications of PRAME expression in KC, it was imperative to study retinoid response in cancer models. Hence, we evaluated whether PRAME modifies the growth‐suppressive effects of retinoids in KC cells. We performed clonogenic assays to study the effect of retinoids upon cell growth/survival. After daily treatments with ATRA or the second‐generation retinoid tazarotene, the number of EV colonies was reduced relative to the number of *PRAME* OE colonies (Fig. 2B–D). Live‐cell proliferation analyses show that while retinoids disrupt the growth of EV cells, the growth of *PRAME* OE cells was minimally affected by retinoid treatment (Fig. 2E, Fig. S2e). These results were reproduced in the UPCI: SCC‐154 cell line (Fig. S2f,g). To further assess whether PRAME attenuates retinoid‐induced growth suppression, we performed Ki‐67 immunofluorescence staining. In agreement with the previous results, the percentage of Ki‐67 positive cells was decreased upon ATRA treatment in EV cells. However, there was not a significant decrease in the *PRAME* OE population (Fig. 2F). Altogether, these results suggest that in KC cells, PRAME modifies the transcriptional response to retinoid treatment, impairing RARE activation and retinoid‐induced growth suppression.

**Fig. 3:**
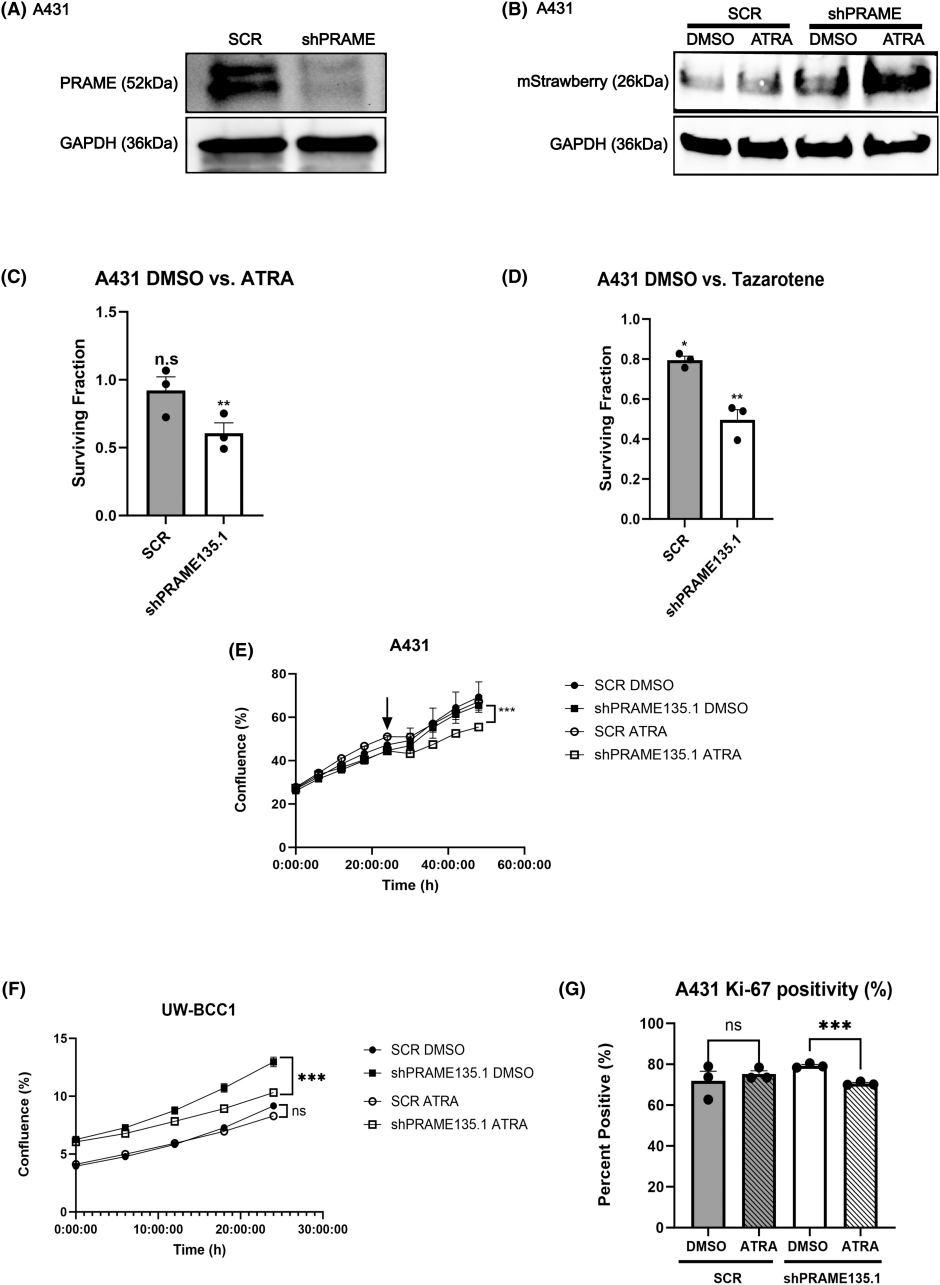
*PRAME* knockdown recaptures retinoid‐induced growth suppression in KC cell lines. (A) Representative western blot for *PRAME* shRNA knockdown (sh*PRAME*135.1) in A431 cells. Scrambled shRNA sequence (SCR) was used as a nonsilencing control. GAPDH was probed as a loading control (*n* = 3). (B) Western blot for mStrawberry in A431 SCR and sh*PRAME*135.1 cells treated with 20 μm ATRA or DMSO. GAPDH was probed as loading control (*n* = 1). Western blot experiments were replicated in multiple cell lines/knockdown clones shown in Fig. S3. (C) Surviving fraction of SCR and sh*PRAME* A431 cells treated with 20 μm ATRA (*n* = 3). DMSO treatment is set as onefold when comparing cell survival in response to drug treatment. Statistical significance determined by applying Student's two‐tailed *t*‐test to colony counts, comparing DMSO to retinoid treatment. (D) Surviving fraction of SCR and shPRAME A431 cells treated with 10 μm tazarotene or DMSO (*n* = 3). DMSO treatment is set as onefold when comparing cell survival in response to drug treatment. Statistical significance determined by applying Student's two‐tailed *t*‐test to colony counts, comparing DMSO to retinoid treatment. (E) IncuCyte® proliferation analysis of SCR and sh*PRAME* A431 cells treated with 20 μm ATRA or DMSO (*n* = 3). Arrow indicates the start of treatment. Two‐way ANOVA. (F) IncuCyte® proliferation analysis of SCR and sh*PRAME* UW‐BCC1 cells treated with 10 μm ATRA or DMSO (*n* = 3). Two‐way ANOVA. (G) Percentage of Ki‐67‐positive cells SCR vs. sh*PRAME* A431 cells treated with 20 μm ATRA or DMSO (*n* = 3). Student's two‐tailed *t*‐test. ns, not significant, *P < 0.05, **P < 0.01, ***P < 0.001. Error bars indicate SEM.

Next, we assessed whether retinoid‐induced transcription was modified by PRAME KO. We treated UW‐BCC1 sgSCR and sg*PRAME* cells with ATRA or DMSO followed by RNA‐Seq analysis. Numerous ATRA‐regulated transcripts are unique to either sgSCR or sg*PRAME* conditions, suggesting altered transcriptional responses to retinoid compounds (Fig. S3a). To isolate the effect of *PRAME* depletion upon retinoid‐induced RARE activation, we performed *PRAME* KD in A431 and UW‐BCC1 cells (Fig. 3A, Fig. S3b–g). *PRAME* KD and SCR control cells were transfected with the RAR‐TRE‐mStrawberry plasmid, and production of the mStrawberry protein was evaluated after ATRA treatment (Fig. 3B, Fig. S3h). SCR cells did not exhibit an increase in mStrawberry production after ATRA treatment, while sh*PRAME* cells exhibited increased baseline production of mStrawberry which was further augmented upon ATRA treatment.

Lastly, we evaluated the effect of *PRAME* KD upon retinoid‐induced growth suppression. We performed clonogenic assay on A431 and UW‐BCC1 *PRAME* KD and SCR control cells, treated with retinoids or DMSO. After daily treatments with ATRA or tazarotene, the surviving fraction of *PRAME* KD cells was lower when compared to SCR control cells (Fig. 3C,D, Fig. S3i). Additionally, upon ATRA treatment, cell proliferation was affected to a greater extent in *PRAME* KD than in SCR cells (Fig. 3E,F, Fig. S3j,k). We further assessed proliferation using Ki‐67 immunofluorescence staining. The percentage of Ki‐67 positive cells was decreased by ATRA treatment in the *PRAME* KD condition, but not in the SCR control condition (Fig. 3G). These results further suggest that in KC cells, PRAME impairs RARE activation and diminishes retinoid‐induced growth suppression.

### 3.3 PRAME prevents retinoids from normalizing keratinization in SCC

**Fig. 4:**
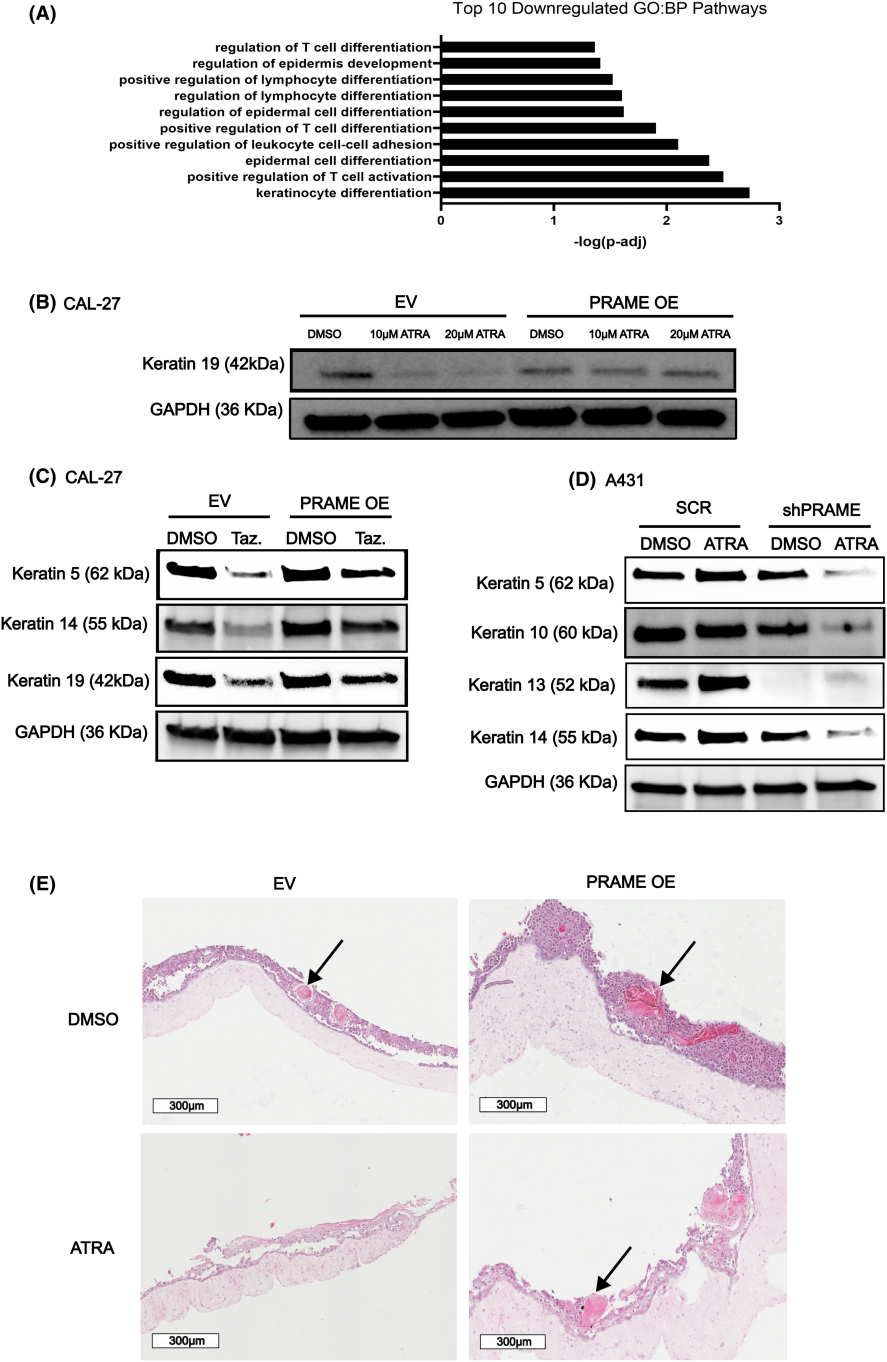
PRAME expression prevents retinoids from normalizing keratinization in SCC. (A) Pathway enrichment graph of genes downregulated upon ATRA treatment in CAL‐27 EV cells (*n* = 3). (B) Western blot for keratin 19 in CAL‐27 EV and *PRAME* OE cells treated with 10 or 20 μm ATRA or DMSO. GAPDH was probed as a loading control (*n* = 1). (C) Western blot for keratins in CAL‐27 EV and *PRAME* OE cells treated with 10 μm tazarotene or DMSO. GAPDH was probed as loading control (*n* = 1). (D) Western blot for keratin expression in A431 SCR and sh*PRAME* cells treated with 20 μm ATRA or DMSO. GAPDH was probed as loading control (*n* = 1). (E) Representative H&E staining of CAL‐27 EV and *PRAME* OE tumoroids, arrows indicate keratin pearls (*n* = 3). 10 μm ATRA used for treatment. Images captured at 10× magnification (Scale bar 300 μm). Western blot results were replicated in multiple cell lines/knockdown clones shown in Fig. S4.

Having demonstrated that PRAME impairs retinoid‐induced growth suppression in KC cells, we aimed to study how PRAME modifies retinoid action in BCC and cSCC. One mechanism by which retinoids suppress SCC pathogenesis is by modulating aberrant keratinocyte/squamous differentiation programs [32]. Specifically, retinoid treatment downregulates the excess keratinization that defines SCC across anatomical sites [32, 33, 34]. Therefore, we assessed the impact of *PRAME* on the ability of retinoids to repress excess keratinization in SCC. RNA‐Seq analyses demonstrated that biological processes downregulated after ATRA treatment in CAL‐27 EV cells (but not in *PRAME* OE cells) include keratinocyte/epidermal differentiation pathways (Fig. 4A). SCC and keratinization‐associated genes in these pathways, such as *NOTCH1, CERS3*, and *AQP3*, are downregulated in EV cells after ATRA treatment. This finding suggests that PRAME impairs the ability of ATRA to repress the inherent overactive SCC‐ and keratinization‐associated gene programs in SCC models.

Next, we studied whether aberrant keratinization may be restrained by retinoid treatment in *PRAME* OE cells. To this end, we conducted western blotting for keratin proteins. In EV cells, SCC‐associated keratin 19 was downregulated in response to ATRA treatment. However, in *PRAME* OE cells this downregulation was not observed (Fig. 4B). Similar patterns were observed when probing for SCC‐associated keratins 5 and 14 (Fig. S4a). Considering that tazarotene is widely appreciated for its antikeratinization activities [98], we treated CAL‐27 cells with this drug. Tazarotene treatment caused a marked and consistent downregulation of keratin 5, 14, and 19, but these effects were attenuated upon *PRAME* OE (Fig. 4C). These results were confirmed using the cancer cell line UPCI:SCC154 and the nontransformed keratinocyte cell lines, HaCaT and Ker‐CT (Fig. S4b–e). Anticipating opposite outcomes, we evaluated how retinoids modulate keratinization in A431 SCR and sh*PRAME* cells. Pronounced repression of keratinization was observed after ATRA treatment in two different *PRAME* KD clones (Fig. 4D, Fig. S4f).

To determine whether these observations translate to changes in tumor keratinization, we constructed tumoroid models composed of normal keratinocytes and SCC cells. Prominent keratin pearls were observed in both the EV and *PRAME* OE SCC tumoroids (Fig. 4E). Daily retinoid treatment made the keratin pearls less prominent. Whereas in *PRAME* OE organoids, this effect was not observed (Fig. 4E). Altogether, the evidence presented indicates that *PRAME* attenuates the ability of retinoids to suppress aberrant keratinization in SCC cells, reducing therapeutic efficacy.

### 3.4 PRAME knockdown restores retinoid‐induced cell death response in BCC cells

**Fig. 5:**
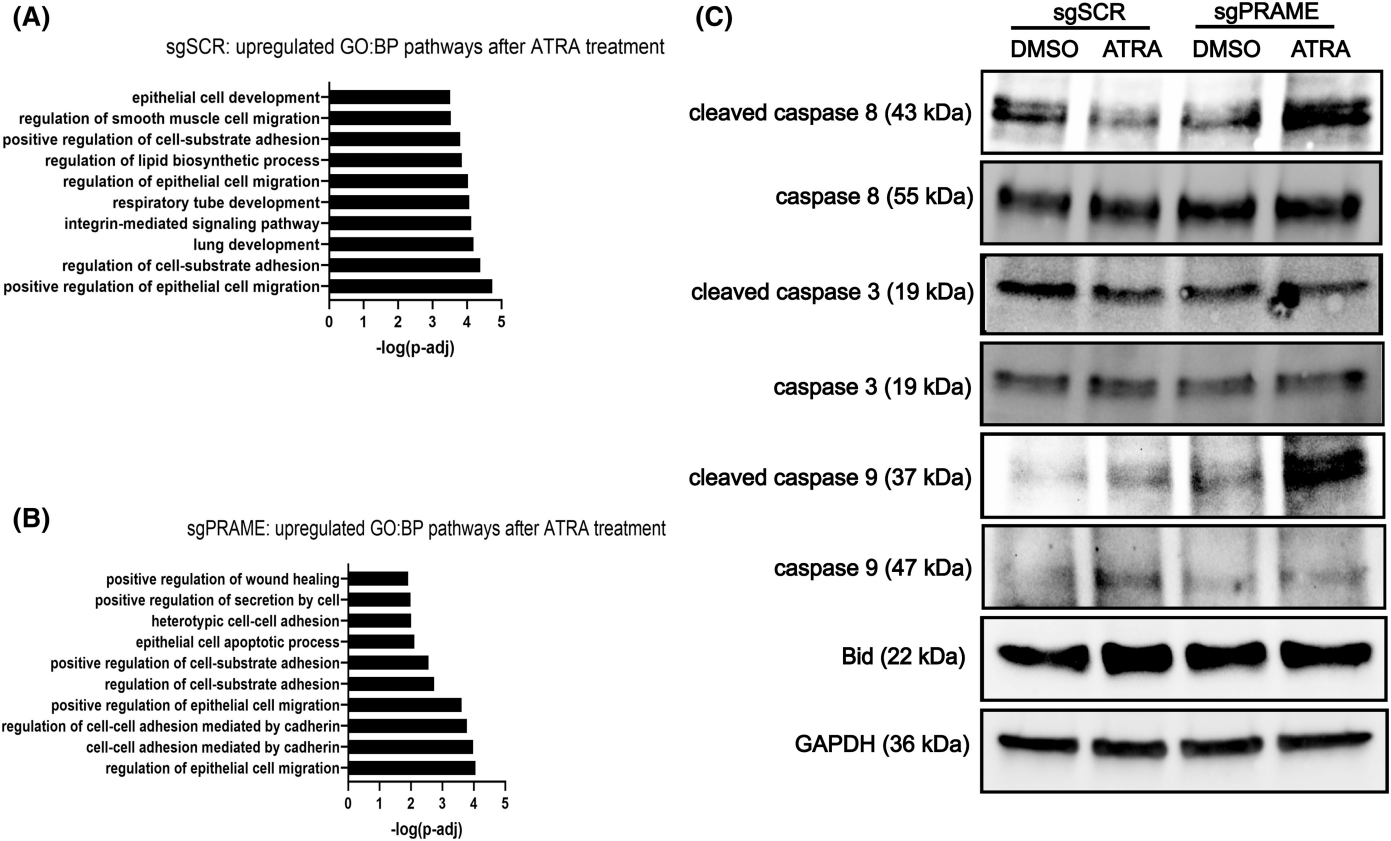
*PRAME* knockdown restores retinoid‐induced cell death response in BCC cells. (A) GO:BP terms for all upregulated genes in UWBCC1 sgSCR cells treated with 10 μm ATRA relative to DMSO (*n* = 3). (B) GO:BP terms for all upregulated genes in UWBCC1 sg*PRAME* cells treated with 10 μm ATRA relative to DMSO. (C) Western blot for apoptotic markers in UW‐BCC1 sgSCR and sg*PRAME* cells treated with 10 μm ATRA or DMSO. GAPDH was probed as a loading control (*n* = 1). Western blot results were replicated in a second knockdown clone shown in Fig. S5.

Normal human keratinocytes and BCC cells respond to retinoids by activating cell death pathways [27, 99, 100]. Interestingly, genes associated with executing cell death programs, like regulated necrosis and proptosis, were enriched in UW‐BCC1 cells after *PRAME* depletion (Fig. S5a). This observation may indicate that these cells are primed to be more responsive to death‐promoting stimuli. ATRA treatment in UW‐BCC1 upregulated the pathway ‘epithelial cell apoptotic process’ in sg*PRAME* exclusively and not in sgSCR control cells (Fig. 5A,B). For example, the proapoptotic *BID* gene was significantly upregulated by ATRA in sg*PRAME* cells, but not sgSCR cells (Fig. S5b). To validate whether retinoid‐induced cell death processes are modified by PRAME expression, we probed for cell death markers after ATRA treatment in sgSCR and sg*PRAME* cells. ATRA treatment increased cleaved caspase 8 and cleaved caspase 9 in sg*PRAME* cells but not in sgSCR control cells (Fig. 5C). Interestingly, *PRAME* KD with shRNA enabled selective increase in cleaved caspase‐8 and the necroptosis marker p‐MLKL after ATRA treatment (Fig. S5c). Apoptotic markers such as cleaved caspase 9 were unchanged after ATRA treatment in the KD model. Overall, these findings suggest that *PRAME* depletion enables ATRA‐induced cell death processes in BCC [101].

### 3.5 EZH2 inhibition overcomes retinoid resistance in PRAME‐expressing BCC and cSCC cells

**Fig. 6:**
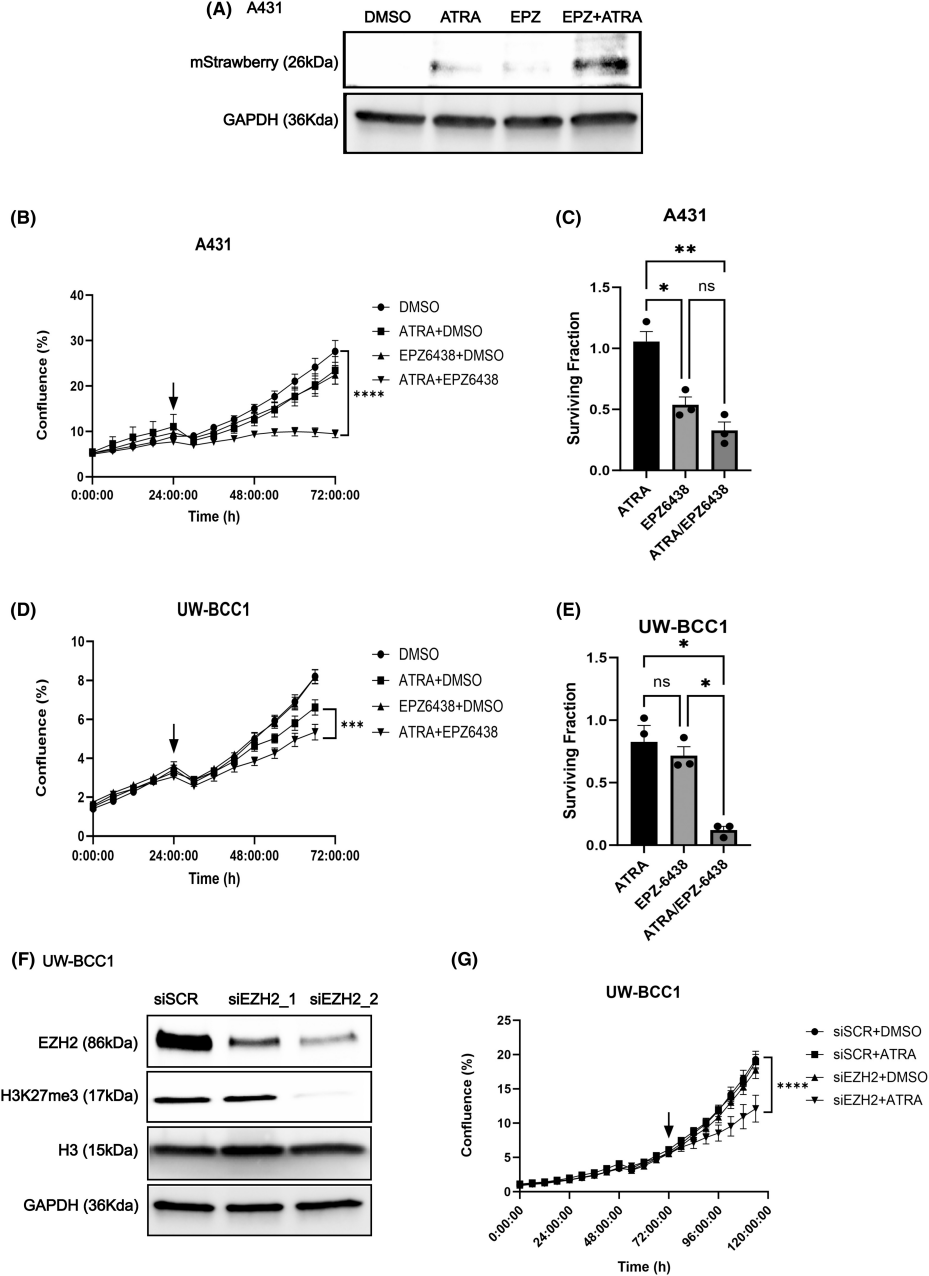
EZH2 inhibition overcomes retinoid resistance in PRAME‐expressing BCC and cSCC cells. (A) Western blot for mStrawberry protein in A431 cells treated with 10 μm ATRA, 20 μm EPZ‐6438, 20 μm EPZ‐6438 + 10 μm ATRA, or DMSO control. GAPDH was probed as a loading control (*n* = 1). Western blot results were replicated in additional cell lines/knockdown clones shown in Fig. S6. (B) IncuCyte® proliferation analysis of A431 cells treated with 10 μm ATRA, 20 μm EPZ‐6438, 10 μm EPZ‐6438 + 20 μm ATRA, or DMSO (*n* = 3). Arrow indicates treatment time. Two‐way ANOVA. (C) Clonogenic assay surviving fraction of A431 cells relative to DMSO controls (*n* = 3). Two‐way ANOVA. (D) IncuCyte® proliferation analysis of UW‐BCC1 cells treated with 10 μm ATRA+ 10 μm DMSO, 10 μm EPZ‐6438 + DMSO, or 10 μm EPZ‐6438 + 10 μm ATRA (*n* = 3) or DMSO. Arrow indicates treatment time. Two‐way ANOVA. (E) Surviving fraction of UW‐BCC1 cells relative to DMSO controls (*n* = 3). Two‐way ANOVA. (F) Western blotting for siRNA mediated *EZH2* knockdown in UW‐BCC1 cells. H3K27me3 levels probed to evaluate functional repression of EZH2 activity. GAPDH and total histone H3 were probed as loading controls (*n* = 1). (G) Incucyte® proliferation analysis of UW‐BCC1 cells treated with DMSO+ 25 nm siSCR, 10 μm ATRA+ 25 nm siSCR, DMSO+ 25 nm si*EZH2*, or 10 μm ATRA+ 25 nm si*EZH2* (*n* = 3). Two‐way ANOVA. ns, not significant, *P < 0.05, **P < 0.01, ***P < 0.001, ****P < 0.0001. Error bars indicate SEM.

Having demonstrated that PRAME attenuates retinoid responses in KC cells, we contemplated strategies to overcome PRAME‐mediated retinoid resistance. EZH2 is the downstream effector arm of PRAME‐mediated retinoid resistance, depositing repressive H3K27me3 to hinder RARE activation [44]. Therefore, we hypothesized that EZH2 inhibition could promote retinoid sensitivity in KC cells that are ectopically expressing PRAME. To evaluate whether combined EZH2 inhibition and retinoid treatment could promote retinoid sensitivity, we first transfected PRAME‐expressing UW‐BCC1 and A431 cells with the RAR‐TRE‐mStrawberry plasmid to evaluate RARE activation. Cells were treated with ATRA, the EZH2 inhibitor EPZ‐6438 (tazemetostat), a combination of both drugs, or with vehicle control (DMSO). We then evaluated mStrawberry production. In A431 cells, combination treatment selectively increased production of mStrawberry, indicating increased RARE activation (Fig. 6A, Fig. S6c). Subsequently, we assessed whether combining retinoid treatment with EZH2 inhibition could suppress growth of PRAME‐expressing KC cells. We treated cells with ATRA, EPZ‐6438, a combination of both drugs or vehicle and performed live‐cell proliferation analyses. Combining ATRA and EPZ‐6438 treatments resulted in greater suppression of cell growth compared to monotherapy (Fig. 6B,D, Fig. S6d,e). These results were validated using clonogenic assays (Fig. 6C,E). Furthermore, these results were reproduced using *EZH2*‐targeting siRNA. *EZH2* silencing combined with ATRA treatment resulted in a greater suppression of cell growth and colony formation in both BCC and cSCC cells, compared to single manipulations (Fig. 6F,G; Fig. S6f–i). Our results indicate that combining EZH2 inhibition with retinoid treatment may be an effective strategy to repress the growth of KC cell lines that express PRAME.

## 4 Discussion

The present study demonstrates that PRAME impairs epidermal differentiation and retinoid response in KC. We also demonstrate that combined application of an EZH2 inhibitor together with a retinoid overcomes PRAME‐mediated retinoid resistance. Therefore, this work proposes that PRAME is a putative biomarker of differentiation and retinoid resistance and is a target to improve retinoid sensitivity in KC. Moreover, this study offers the EZH2 inhibitor/retinoid combination as a compelling drug repurposing strategy to manage KC. The insights provided herein may help optimize the use of retinoids for KC prevention and treatment and may eventually help reduce the need for surgical interventions to manage KCs.

Consistent with a report from other cancers, we demonstrate that PRAME regulates cell differentiation in KC [53]. Terminal differentiation and keratinization pathways that are normally downregulated in BCC [17], are restored upon *PRAME* KO. Conversely, these pathways are downregulated upon *PRAME* OE. Moderate‐ to poorly differentiated cSCC, representing aggressive disease, exhibits higher PRAME expression compared to well‐differentiated cSCC [60]. Thus, PRAME may represent a biomarker for KC differentiation and may associate with aggressive disease. Transcriptomic analyses also illustrated a negative correlation between PRAME expression and interferon signaling. A link between PRAME and interferon signaling pathways has been suggested, although never demonstrated in molecular studies [102]. Some targets and coregulators of PRAME's activity in cancer (e.g., p14/ARF, EZH2) are mediators of immunological signaling [103, 104, 105]. Additionally, interactions between retinoid and interferon signaling pathways have been documented and exploited in clinical studies [106, 107]. Considering the role of immunological signaling in KC pathogenesis and potential implications for immunotherapy, studies probing a possible relationship between PRAME and interferon signaling may be informative [108].

Growth‐suppressive retinoid responses were attenuated by *PRAME* OE and restored by *PRAME* downregulation in BCC and SCC cells. These findings are consistent with reports illustrating the ability of PRAME to regulate retinoid response in other types of cancer [109, 110]. Retinoids are common treatments for skin diseases and have notable effects upon BCC and cSCC [21]. Therefore, our study evaluates PRAME‐mediated retinoid resistance in this uniquely relevant context. Having also observed PRAME expression in actinic keratoses, we propose that PRAME may also influence retinoid‐based chemoprevention.

We have determined that PRAME prevents retinoids from downregulating aberrant keratinization in SCC cells. To some, this finding would appear to contradict the earlier assertion that PRAME impairs keratinocyte differentiation gene signatures. However, these findings should be considered independently. First, the effect of PRAME on differentiation was studied in the absence of exogenous retinoid treatment. Likewise, the negative correlation between PRAME and differentiation in SCC tumor tissues was also observed independent of prior retinoid treatment. Additionally, while PRAME downregulated keratinocyte differentiation signatures in normal keratinocyte and BCC cell lines, PRAME OE had no impact on keratinocyte differentiation gene expression program in the CAL‐27 cell line. Nonetheless, PRAME OE rendered this cell line unresponsive to retinoid‐induced repression of keratinization. Hence, PRAME's effect on differentiation may be independent of retinoid signaling and may relate to one of PRAME's other cellular activities, and subject to influence by intrinsic differentiation aberrations specific to cell lines/tumor type [57, 58]. Further studies to uncover this relationship are warranted.

This study demonstrates that PRAME‐mediated retinoid resistance could be overcome by targeting EZH2. Epigenome‐modifying drugs and retinoids have been combined to treat cancer before [22]. Vorinostat, a histone deacetylase (HDAC) inhibitor, has been used with a rexinoid (RXR‐targeting retinoid) to treat cases of cutaneous T‐cell lymphoma [111]. Another study used an HDAC inhibitor to bypass PRAME‐mediated retinoid resistance in breast cancer cells [112]. EZH2 has been contemplated as a therapeutic target for several types of cancer, including BCC and cSCC [113, 114, 115]. The recent adoption of EZH2 inhibitors into clinical practice has motivated efforts to study EZH2 inhibitors in cancer [116]. Recent studies from bladder cancer and rhabdomyosarcoma demonstrated the efficacy of combining certain EZH2 inhibitors with retinoid compounds [117, 118]. To our knowledge, our study is the first to evaluate the EZH2 inhibitor EPZ‐6438 as a treatment for BCC and cSCC, and it is the first to use EZH2 inhibition to address retinoid resistance in KC.

Several other clinically relevant results emerge from this study. First, PRAME can be validated empirically as a biomarker of tumor grade or retinoid response in KC tumors. In addition, studying how PRAME regulates differentiation and interferon signaling may offer insights into PRAME biology and may translate to clinical advances. Lastly, since the mechanism by which the EZH2 inhibitor/retinoid combination can suppress KC progression may be independent of PRAME's activity, further mechanistic characterization may be informative. It is also of vital importance that *in vivo* validation of the findings documented in the present study be conducted using animal models.

## 5 Conclusion

To conclude, this study proposes that PRAME limits the efficacy of retinoids as KC treatments. We propose that overcoming PRAME‐mediated retinoid resistance directly or through EZH2 inhibition may be a viable treatment strategy. Topical and systemic retinoids are widely available, effective, and well‐tolerated drugs used in dermatology that are used for skin cancer prevention and treatment. We propose revisiting the use of retinoids for KC, taking into consideration the impact of PRAME expression. Insights into retinoid resistance mechanisms in KC and new combination treatment strategies may expand the therapeutic use of these drugs, alleviating the dependence upon surgical interventions.

## Conflict of interest

The authors declare no conflict of interest.

## Author contributions

IVL supervised the study, participated in experimental design, provision of resources, methodology, data analysis, interpretation, and data visualization. BR designed and performed experiments, methodology, analyzed primary data, interpretation, data visualization, prepared figures, and wrote the manuscript. AML, SAS, JG, SS, and PL assisted with experiments and contributed to data analysis. BR, IVL, AMV, SAS, and PL edited the manuscript. All authors have read and agreed to the final published version of the manuscript.

## Supporting information


**Fig. S1.** PRAME impairs epidermal differentiation gene signatures in keratinocytes and keratinocyte carcinoma cells.
**Fig. S2.** PRAME impairs retinoid‐induced growth suppression in KC cell lines.
**Fig. S3.** *PRAME* knockdown recaptures retinoid‐induced growth suppression in KC cell lines.
**Fig. S4.** PRAME expression prevents retinoids from normalizing keratinization in SCC.
**Fig. S5.** *PRAME* knockdown restores retinoid‐induced cell death response in BCC cells.
**Fig. S6.** EZH2 inhibition overcomes retinoid resistance in PRAME‐expressing BCC and cSCC cells.

**Fig. S7.** Full uncropped western blot images.

**Table S1.** Patient‐derived skin tumor age, diagnosis and sex.
**Table S2.** *PRAME* shRNA and control shRNA catalogue numbers, sequences and lot numbers.
**Table S3.** *PRAME* sgRNA and control sgRNA catalogue numbers, target sequences and target region.
**Table S4.** Information regarding PRAME ORF overexpression lentiviral particles and control particles.

## Data Availability

The RNA‐Seq data generated in this study has been deposited in the Gene Expression Omnibus (GEO) database accession number: GSE271108.
