# Supplementary material for: Targeting PRAME directly or via EZH2 inhibition overcomes retinoid resistance and represents a novel therapy for keratinocyte carcinoma
Source: Mol Oncol. 2025 Mar 18;19(5):1471–92. doi: 10.1002/1878-0261.13820 (PMC12077289; doi:10.1002/1878-0261.13820)
Supplement: Supplementary file 1 — Fig. S1. PRAME impairs epidermal differentiation gene signatures in keratinocytes and keratinocyte carcinoma cells. Fig. S2. PRAME impairs retinoid‐induced growth suppression in KC cell lines. Fig. S3. PRAME knockdown recaptures retinoid‐induced growth suppression in KC cell lines. Fig. S4. PRAME expression prevents retinoids from normalizing keratinization in SCC. Fig. S5. PRAME knockdown restores retinoid‐induced cell death response in BCC cells. Fig. S6. EZH2 inhibition overcomes retinoid resistance in PRAME‐expressing BCC and cSCC cells. [file MOL2-19-1471-s002.docx]

**Title:** **Targeting PRAME directly or via EZH2 inhibition overcomes retinoid resistance and represents a novel therapy for keratinocyte carcinoma**

**Supplementary Figures:**


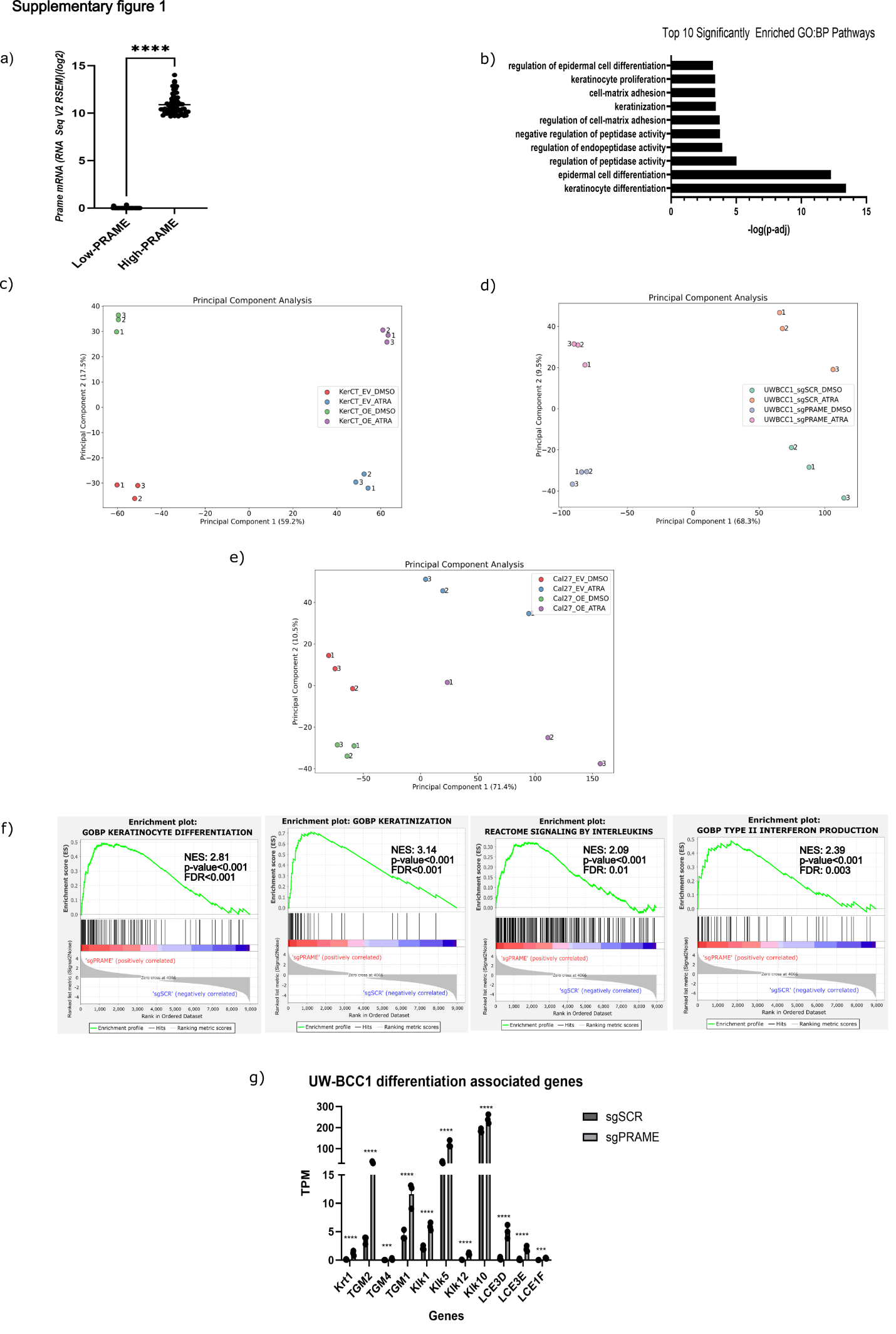


**
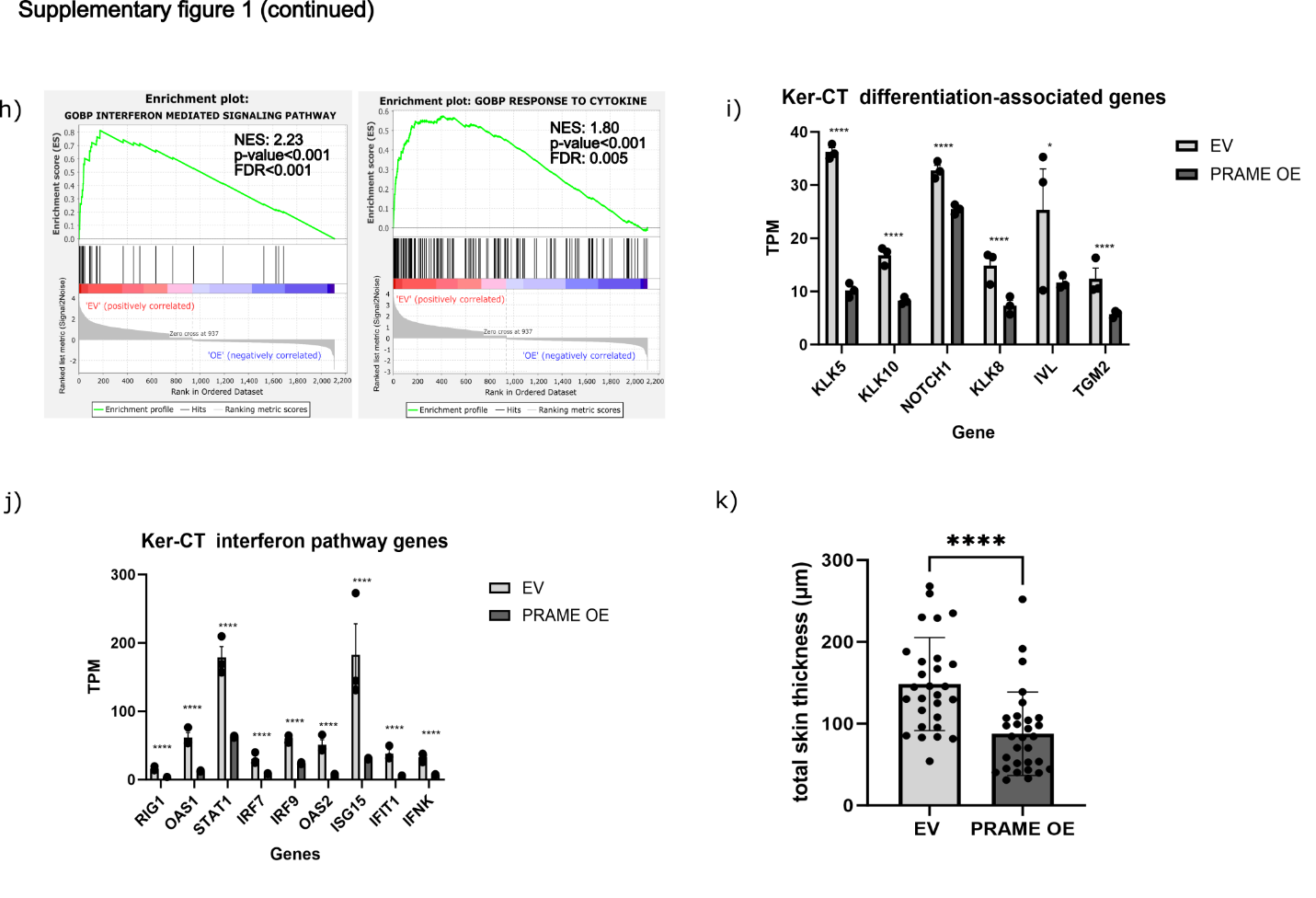
**

**Supplementary Figure S1**. a) PRAME mRNA expression (RNA-seq RSEM V2 log(2)) in the low (n=37) *vs*. high (n=55) *PRAME* expressing cervical SCC (cvSCC) tumors based on data from TCGA. Significance determined using student’s two-tailed t-test. b) GO:BP pathway enrichment bar graph for genes that are significantly enriched in the low- *vs.* the high-*PRAME* expressing groups. c) Principal component analysis (PCA) of RNA-Seq data from Ker-CT EV DMSO, Ker-CT *PRAME* OE DMSO, Ker-CT EV ATRA and Ker-CT *PRAME* OE ATRA. d) PCA of RNA-Seq data from UW-BCC1 sgSCR DMSO, UW-BCC1 sgSCR ATRA, UW-BCC1 sgPRAME DMSO, UW-BCC1 sgPRAME ATRA. e) PCA of RNA-seq data from CAL-27 EV DMSO, CAL-27 EV ATRA, CAL-27 PRAME OE DMSO, CAL-27**. f) Enrichment plots comparing GO:BP pathways enriched in sg*PRAME* vs sgSCR UW-BCC1 cells (n=3). Normalized enrichment score (NES), p value and false discovery rate (FDR) included). g) Enrichment plots comparing top GO:BP pathways enriched in EV vs OE KerCT cells (n=3). NES, p value and FDR included.** h) Transcripts per million (TPM) of selected epidermal-differentiation associated genes in UW-BCC1 sgSCR and sg*PRAME* cells (n=3). i) TPM of selected epidermal differentiation-associated genes in Ker-CT EV and *PRAME* OE cells (n=3). j) TPM of selected interferon signalling genes in Ker-CT EV and *PRAME* OE cells (n=3). k) Total skin thickness of Ker-CT EV and Ker-CT *PRAME* OE organoids. Significance determined using Welch’s t-test. *p<0.05, ***p<0.001, ****p<0.0001. Error bars indicate SEM.


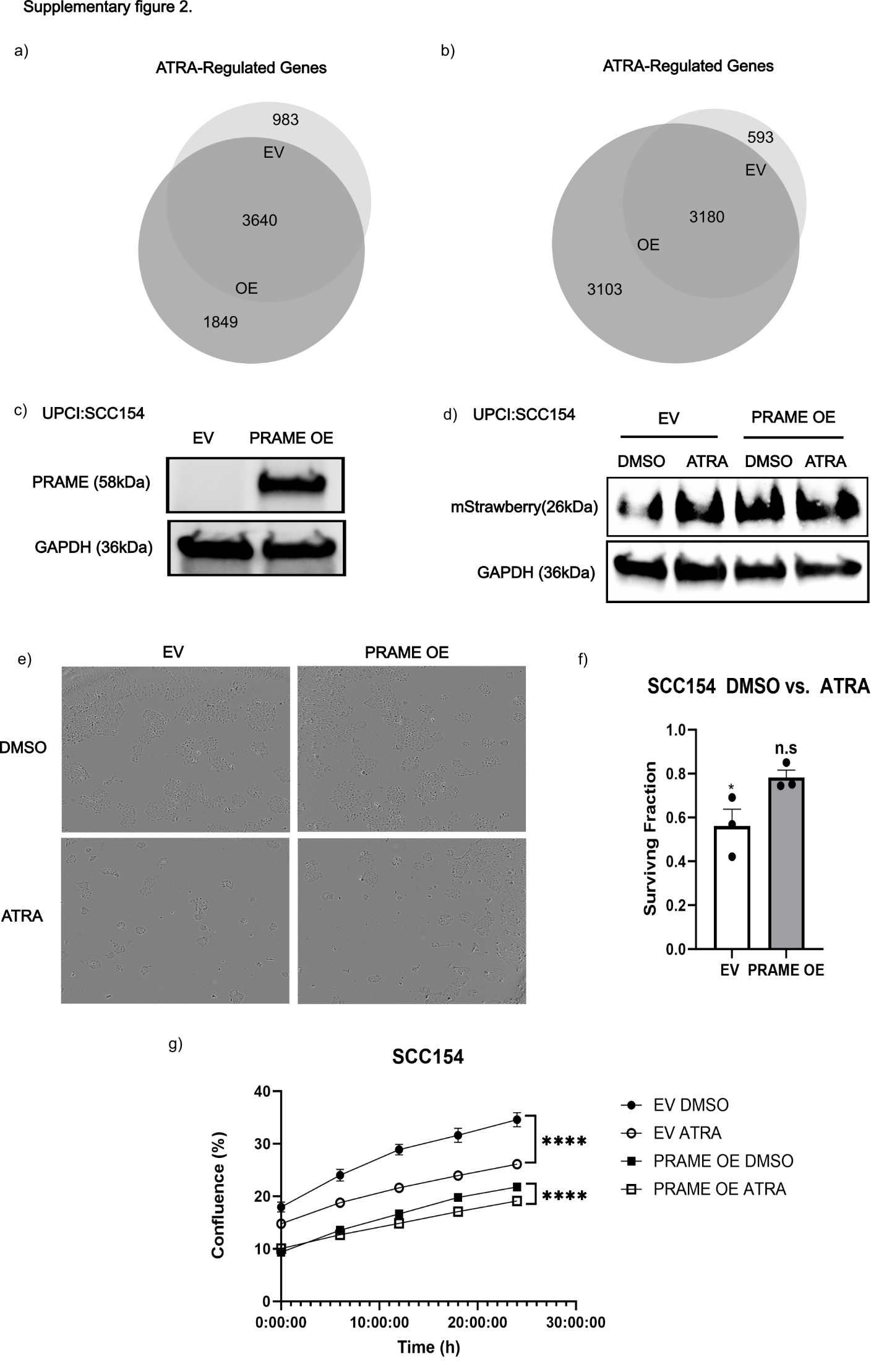


**Supplementary Figure S2**. a) Venn-diagram depicting significant (p<0.05) ATRA-regulated genes in Ker-CT EV and *PRAME* OE cells (n=3). b) Venn diagram depicting significant ATRA-regulated genes in CAL-27 EV and *PRAME* OE cells (n=3). c) Western blot for PRAME in EV or *PRAME* OE UPCI:SCC-154 cells. GAPDH was probed as a loading control. d) Western blot for mStrawberry in UPCI-SCC-154 EV and *PRAME* OE cells transfected with the RAR-TRE-mStrawberry plasmid and treated with 10µM of ATRA or DMSO. GAPDH was probed as a loading control. e) **Representative IncuCyte® images of CAL-27 EV and PRAME OR cells treated with DMSO or 20μM ATRA, taken at 72 hours** f) Surviving fraction of EV and *PRAME* OE UPCI:SCC154 cells treated with ATRA or DMSO. DMSO treatment is set as 1-fold when comparing cell survival in response to drug treatment. Statistical significance determined by applying student’s two-tailed t-test to colony counts, comparing DMSO to retinoid-treated conditions. g) IncuCyte® proliferation analysis of EV and *PRAME* OE UPCI:SCC154 cells treated with 10µM ATRA or DMSO over 24 hours. Two-way ANOVA. n.s not significant, *p<0.05, ****p<0.0001. Error bars indicate SEM.


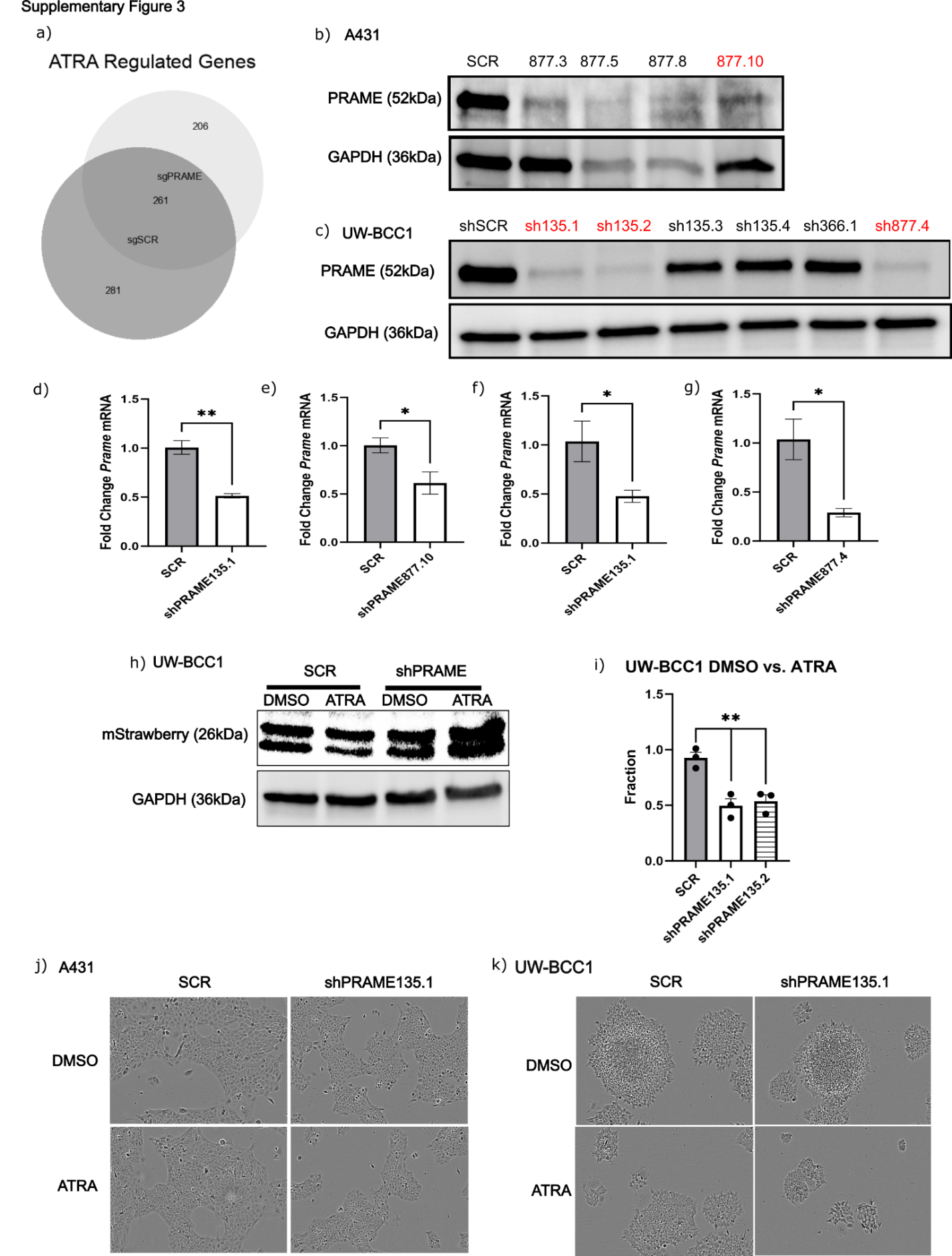


**
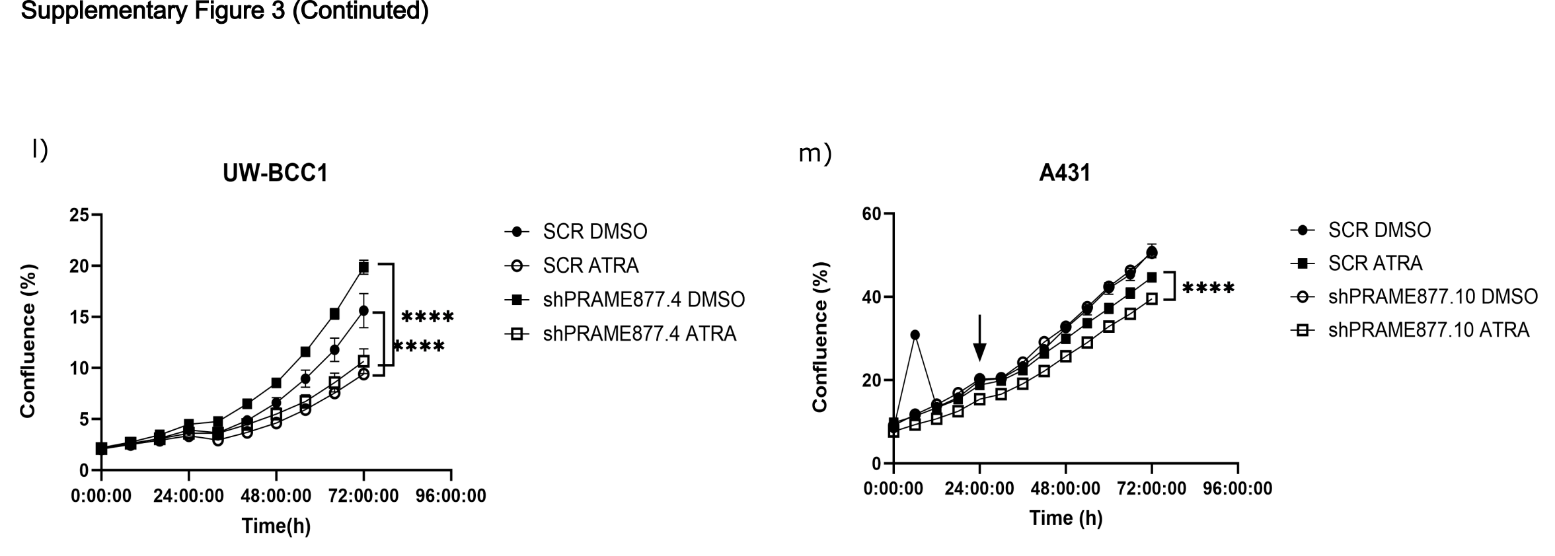
**

**Supplementary Figure S3**. a) Venn diagram depicting significant ATRA-regulated genes in UW-BCC1 sgSCR and sg*PRAME* cells (n=3). b) Western blot for *PRAME* shRNA knockdown clones in A431 cells. Scrambled shRNA sequence (SCR) was used as a non-silencing control. Red font indicates knockdown clones used for subsequent experiments (877.10). GAPDH was probed as a loading control c) Western blot for PRAME shRNA knockdown clones in UW-BCC1 cells. Scrambled shRNA sequence (SCR) was used as a non-silencing control. Red font indicates successful knockdown clones (135.1, 135.2, 877.4). GAPDH was probed as a loading control d) Fold change *PRAME* mRNA in A431 SCR *vs.* sh*PRAME*135.1 cells (n=3). Normalized to *B2M.*  e) Fold change *PRAME* mRNA in A431 SCR and sh*PRAME877*.10 cells (n=3). Normalized to *B2M.* f) Fold change in *PRAME* mRNA in UW-BCC1 SCR and sh*PRAME135*.1 cells. Normalized to *B2M* (n=3) g) Fold change *PRAME* mRNA in UW-BCC1 SCR and shPRAME877.4 cells (n=3). Normalized to *B2M.* h) Western blot for mStrawberry in UW-BCC1 SCR and sh*PRAME* treated with 10µM ATRA or DMSO. GAPDH probed as a loading control. i) Surviving fractions of SCR, sh*PRAME*135.1 and sh*PRAME*135.2 UW-BCC1 cells treated with 10µM ATRA or DMSO (n=3). j) **Representative IncuCyte® images of A431 SCR and shPRAME135.1 cells treated with DMSO or 10μM ATRA taken at 48 hours. k) Representative IncuCyte® images of UW-BCC1 cells treated with DMSO or 10μM ATRA, taken at 24 hours.** l) IncuCyte® proliferation analysis of SCR and sh*PRAME*877.4 UW-BCC1 cells treated with 10µM ATRA or DMSO. DMSO treatment is set as 1-fold when comparing cell survival in response to drug treatment. Statistical significance determined by applying student’s two-tailed t-test to raw colony counts, comparing DMSO to retinoid treated conditions (n=3). Two-way ANOVA. m) IncuCyte® proliferation assay for SCR and sh*PRAME*877.10 A431 cells. Two-way ANOVA. *p<0.05, **p<0.01, ****p<0.0001. Error bars indicate SEM.


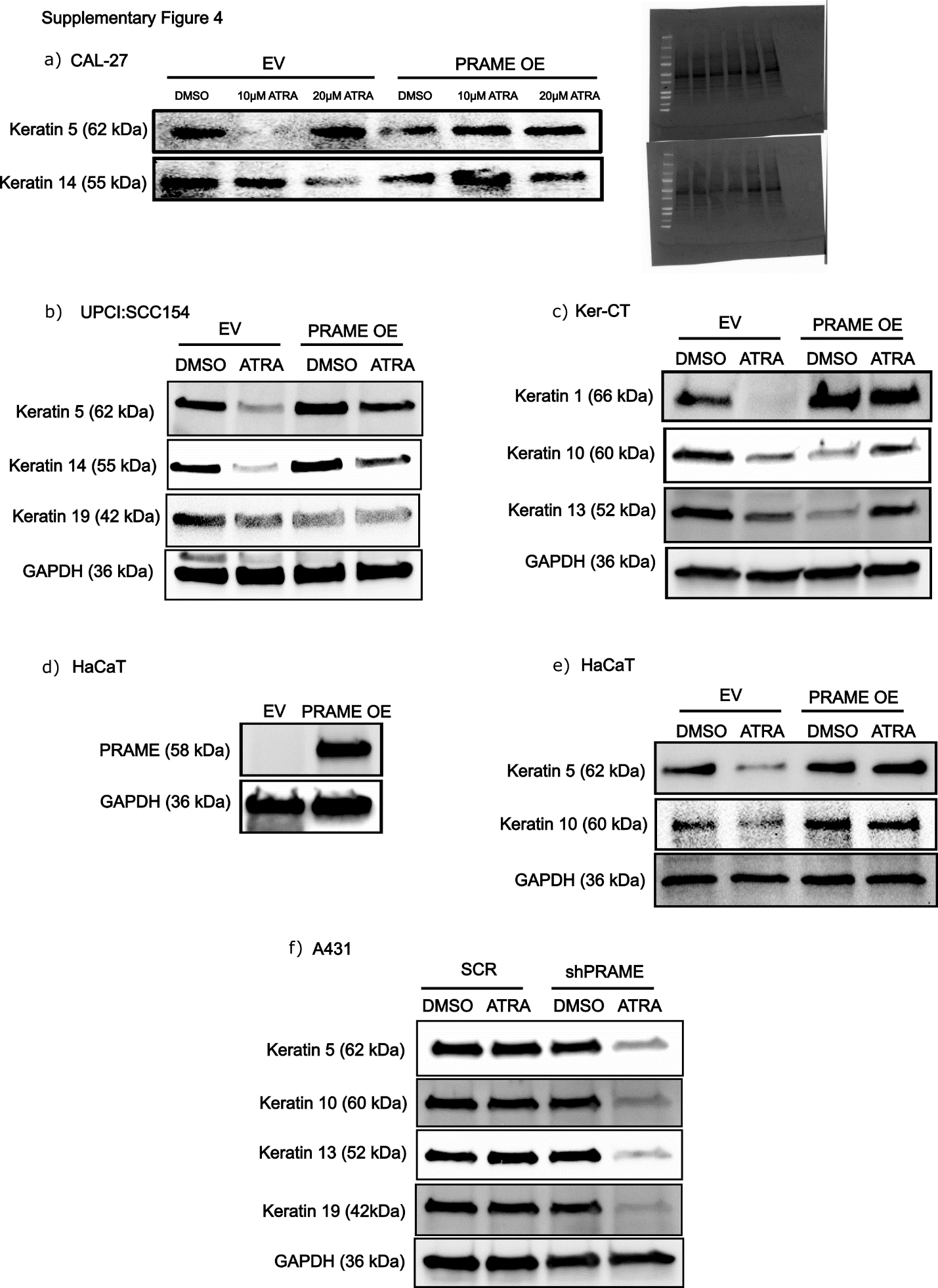


**Supplementary Figure S4**. a) Western blot for cytokeratins in CAL-27 EV and PRAME OE cells treated with DMSO, 10µM or 20µM ATRA for 24 hours. Stain free blots for keratin 14 blot (top) and keratin 5 blot (bottom) pictured on the right as loading controls. b) Western blot for keratin 5, 14 and 19 in UPCI:SCC154 EV and *PRAME* OE cells treated with DMSO or 10µM ATRA for 24 hours. GAPDH was probed as loading control. c) Western blot for keratin 1, 10 and 13 in Ker-CT EV and *PRAME* OE cells treated with DMSO or 2µM ATRA for 24 hours. GAPDH was probed as a loading control. d) Western blot for PRAME in HaCaT EV and *PRAME* OE cells. GAPDH probed as loading control. e) Keratin 5 and 10 in HaCaT EV and *PRAME* OE cells treated with DMSO or 10µM ATRA after 24 hours. GAPDH was probed as a loading control. f) Western blot for cytokeratin 5, 10, 13 and 19 in A431 SCR and sh*PRAME*877.10 cells treated with DMSO or 10µM ATRA for 24 hours. GAPDH was probed as a loading control.


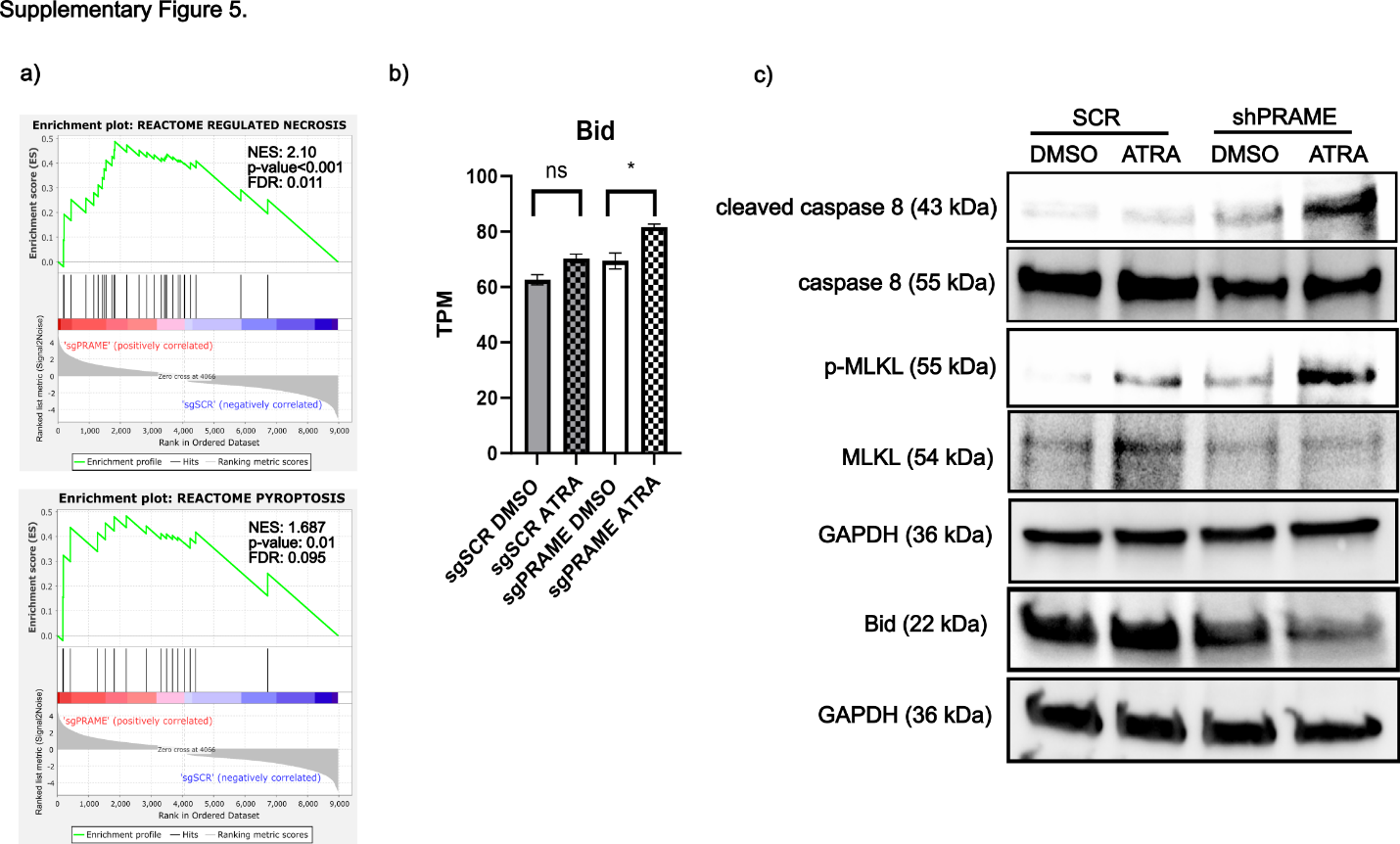


**Supplementary Figure S5**. a) **Enrichment plots comparing cell death associated REACTOME pathways enriched in sg*PRAME* vs sgSCR UW-BCC1 cells (n=3). Normalized enrichment score (NES), p value and false discovery rate (FDR) included.** b) Relative *BID* mRNA expression in Transcripts Per Million (TPM) in UW-BCC1 cells treated with DMSO or 10µM ATRA n.s. non-significant *p<0.05 by DESeq2 pipeline. c) Western blot for the cell death (cleaved-caspase 8) and necroptosis markers (phosphorylated-mixed lineage kinase like (p-MLKL)) in SCR and sh*PRAME*135.1 UW-BCC1 cells treated with DMSO or 10µM ATRA for 24 hours. GAPDH was probed as a loading control. Error bars indicate SEM.


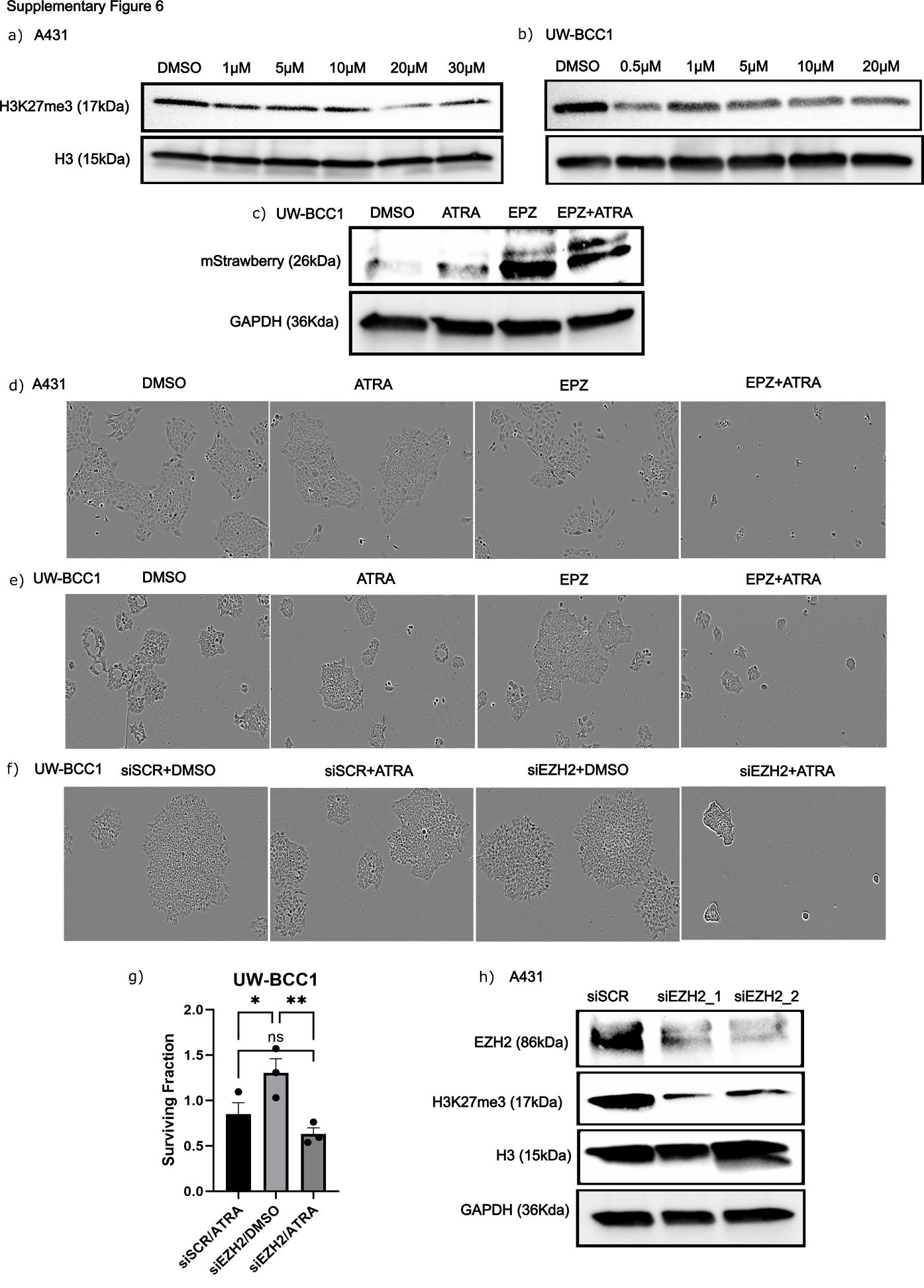


**
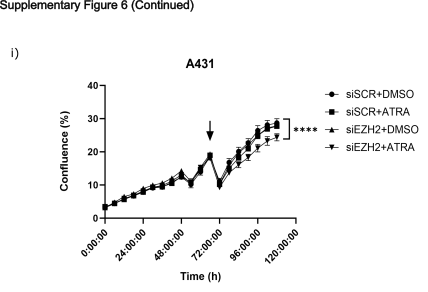
**

**Supplementary Figure S6**. a) Western blot for H3K27me3 levels across different doses of tazemetostat (EPZ-6438) in A431 cells. Total histone H3 probed as loading control. b) Western blot for H3K27me3 levels across different doses of tazemetostat (EPZ-6438) in UW-BCC1 cells. Total histone H3 probed as loading control. c) mStrawberry western blot in UW-BCC1 cells treated with DMSO, 10µM ATRA+DMSO, 20µM EPZ-6438+DMSO, or 10µM ATRA + 20µM EPZ-6438. GAPDH probed as a loading control. d) **Representative IncuCyte images of A431 cells treated with DMSO, 10µM ATRA+DMSO , 20µM EPZ-6438+DMSO, 10µM EPZ-6438+20µM ATRA. Taken at 72 hours. e) Representative IncuCyte images of UW-BCC1 cells treated with DMSO, 10µM ATRA+ 10µM DMSO, 10µM EPZ-6438+DMSO or 10µM EPZ-6438 + 10µM ATRA. Taken at 72 hours.** f) **Representative IncuCyte images of UW-BCC1 cells treated with scrambled 25nM siRNA (siSCR)+10µM ATRA, 25nM si*EZH2*+DMSO, 25nM si*EZH2*+10µM ATRA, relative to control (25nM siRNA+DMSO. Taken at 114 hours.** g) Clonogenic survival of UW-BCC1 cells treated with scrambled 25nM siRNA (siSCR)+10µM ATRA, 25nM si*EZH2*+DMSO, 25nM si*EZH2*+10µM ATRA, relative to control (25nM siRNA+DMSO) (n=3). Two-way ANOVA. h) Western blot for EZH2 in A431 cells treated with 35nM of *EZH2* siRNA. H3K27me3 probed for functional assessment. Total histone H3 and GAPDH probed as loading controls. i) IncuCyte® proliferation analysis of A431 cells treated with 35nM siSCR+ DMSO, 35nM siSCR+ 20µM ATRA, 35nM si*EZH2* + DMSO, 35nM si*EZH2* + 20µM ATRA (n=3). Two-way ANOVA. *p<0.05, **p<0.01, ****p<0.0001. Error bars indicate SEM.
