# Supplementary figures and images for: Targeting PRAME directly or via EZH2 inhibition overcomes retinoid resistance and represents a novel therapy for keratinocyte carcinoma
Source: Mol Oncol. 2025 Mar 18;19(5):1471–92. doi: 10.1002/1878-0261.13820 (PMC12077289; doi:10.1002/1878-0261.13820)

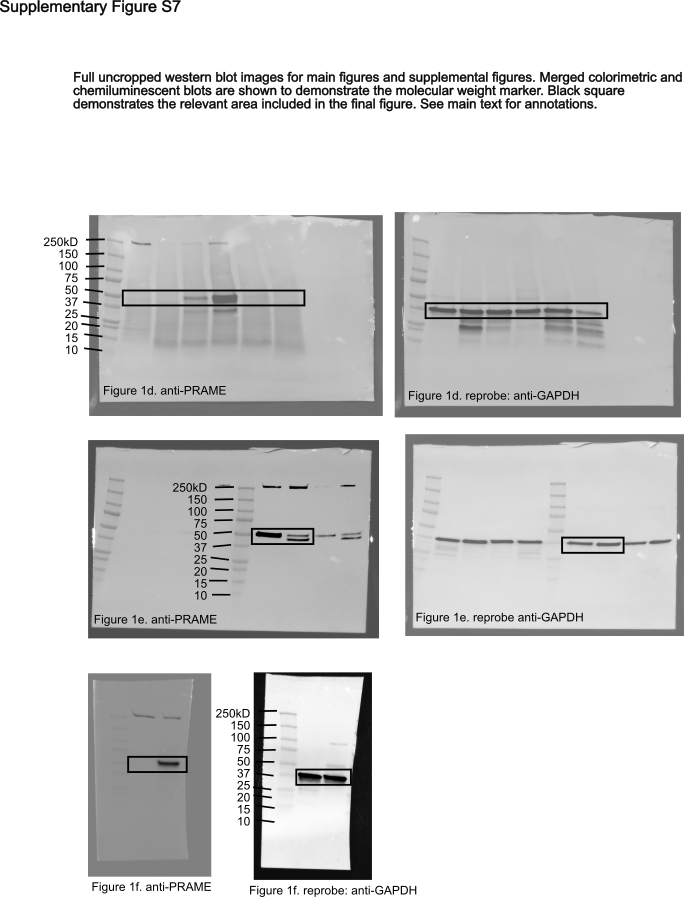


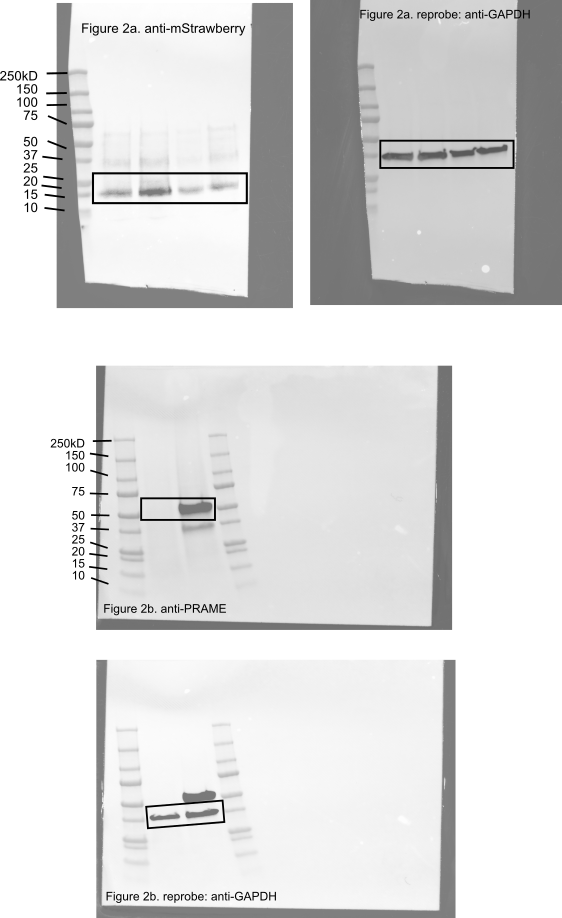


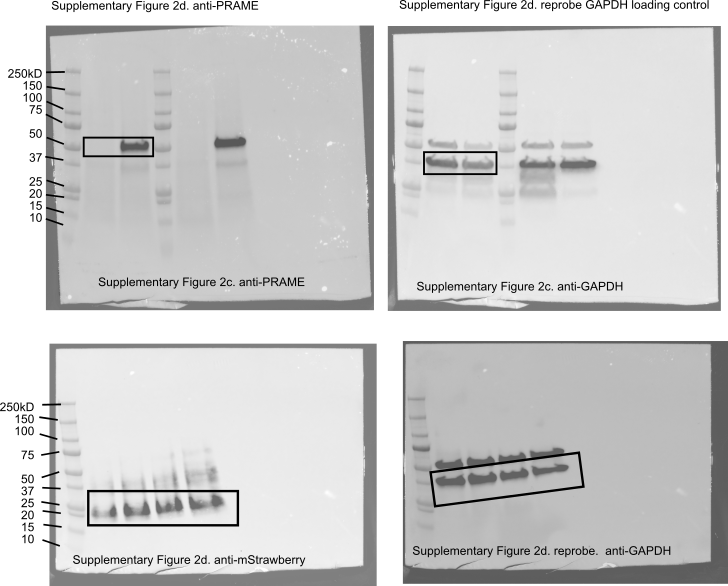


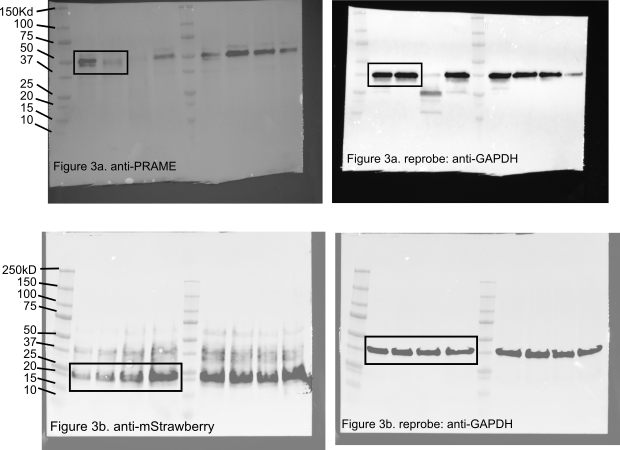


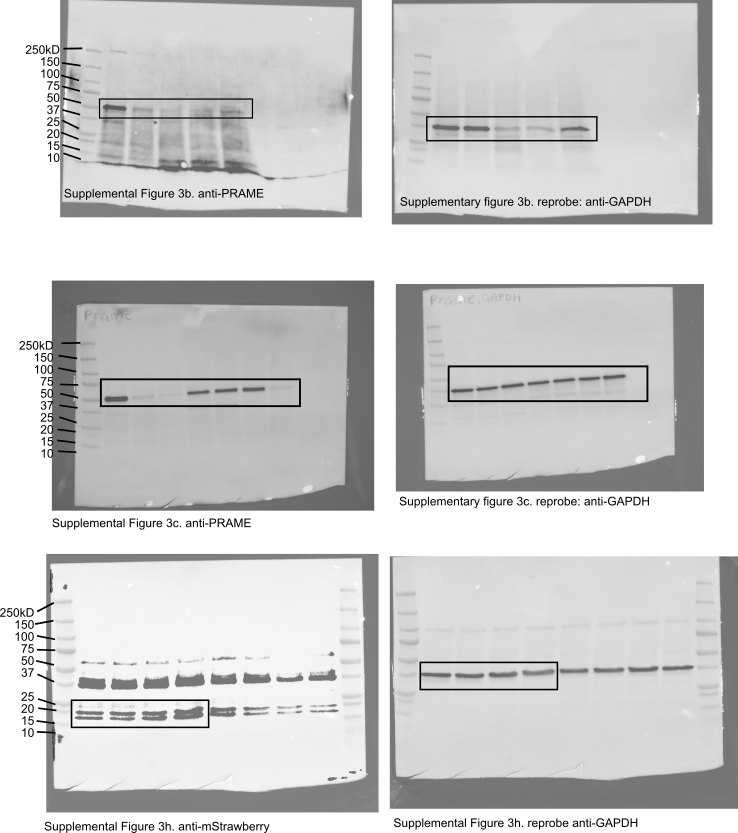


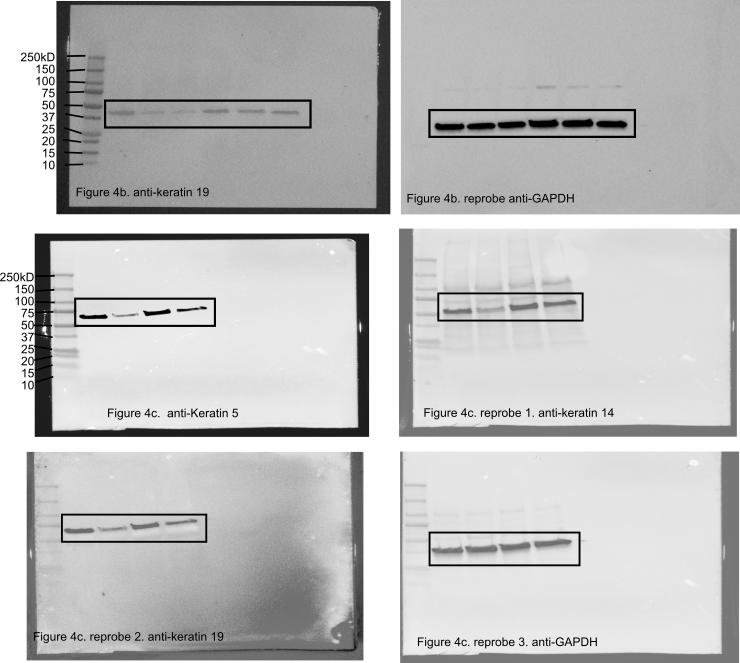


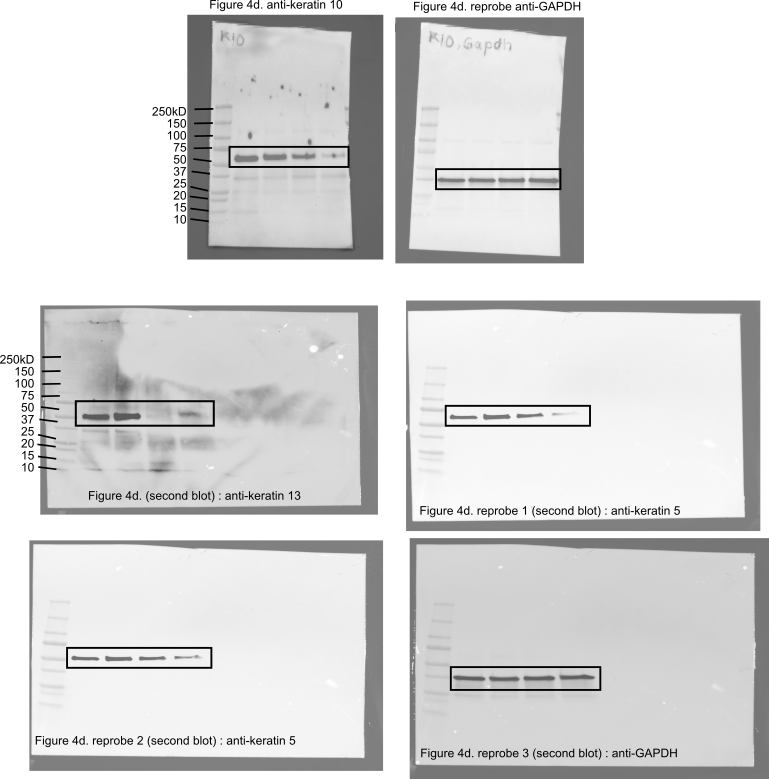


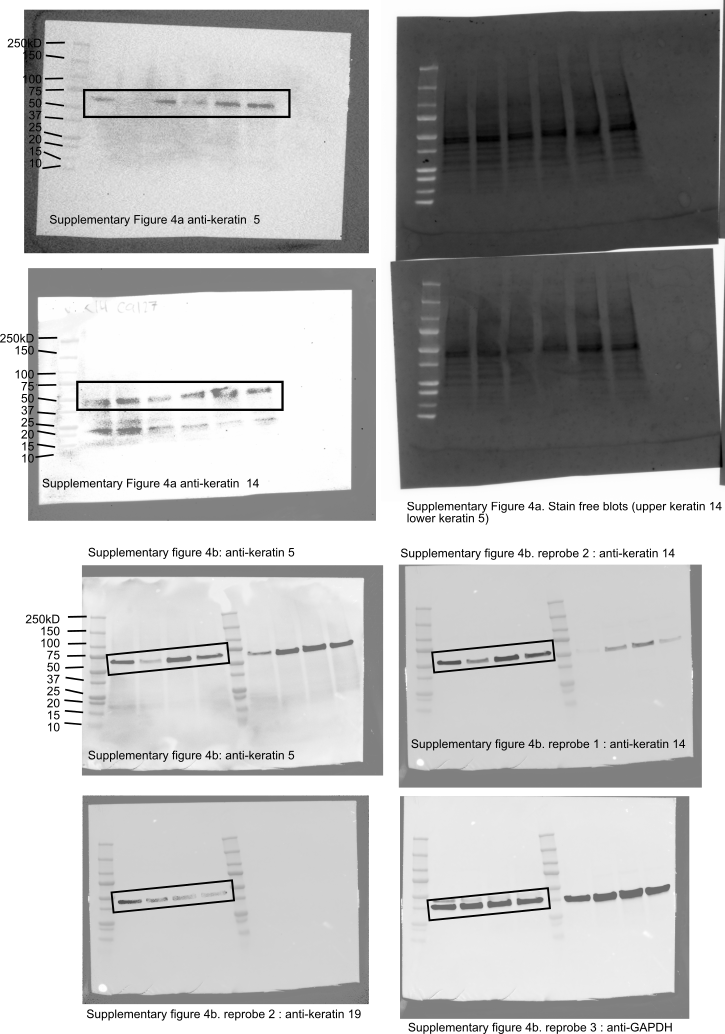


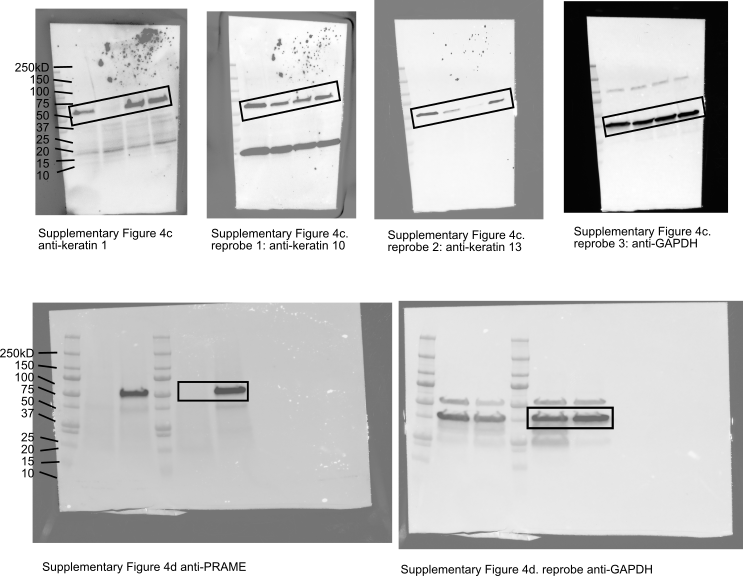


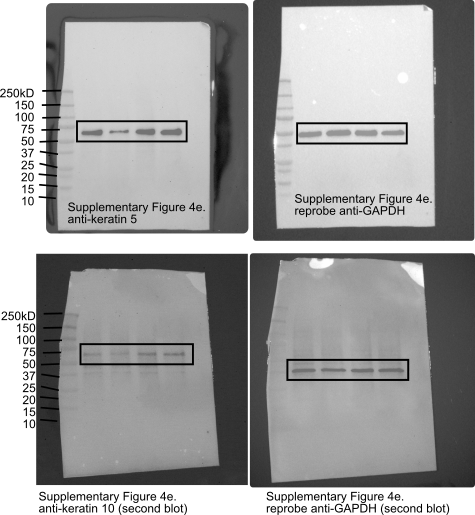


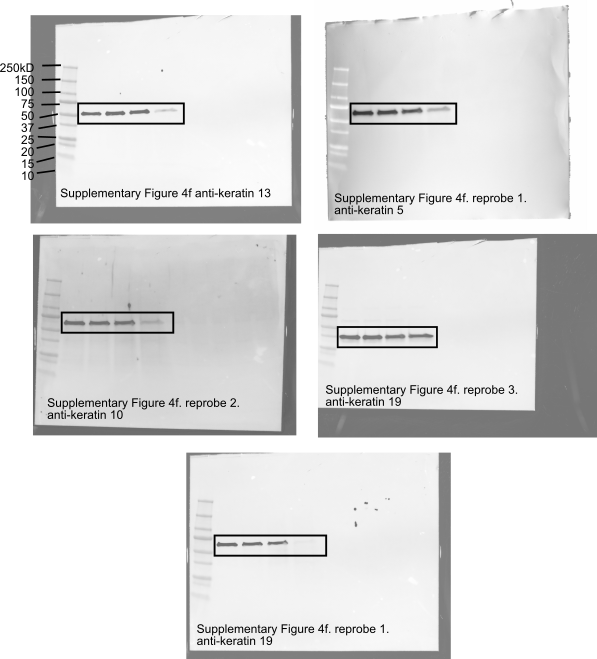


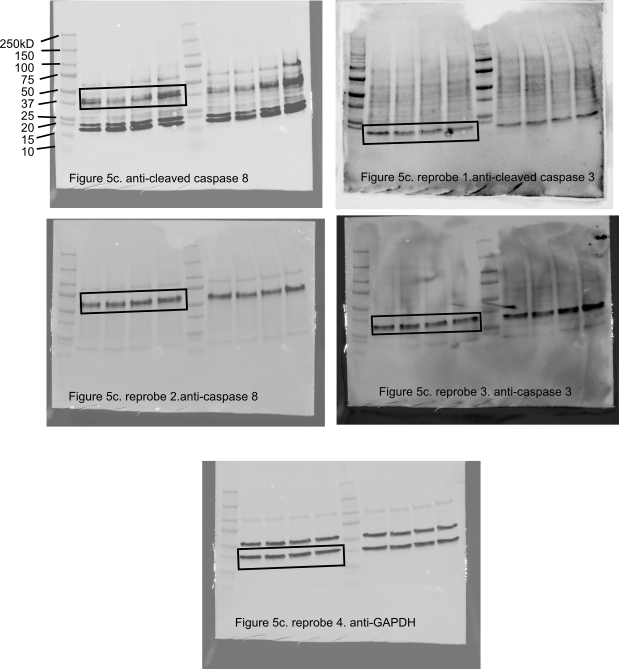


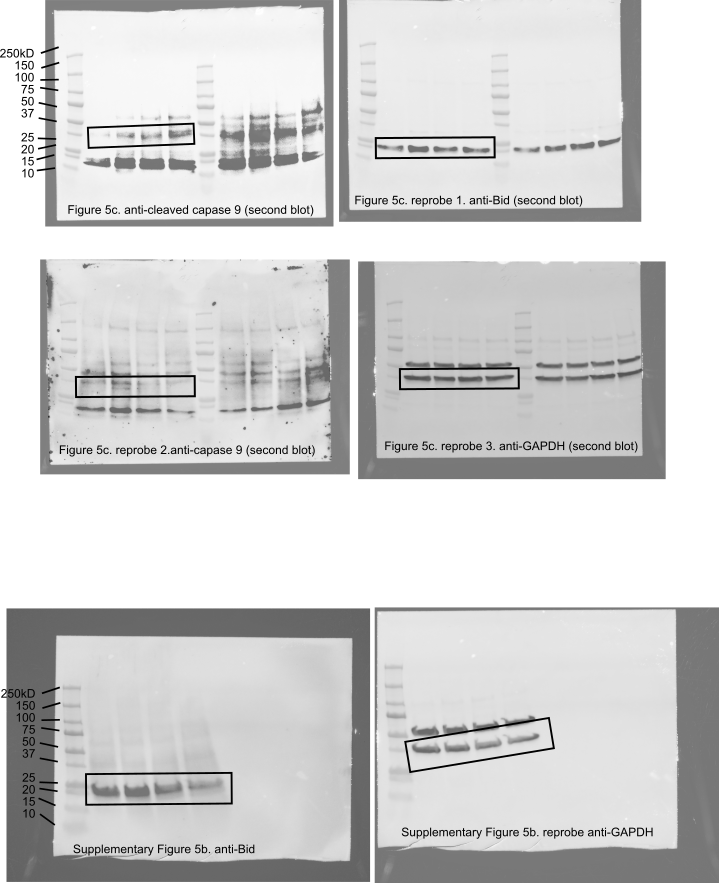


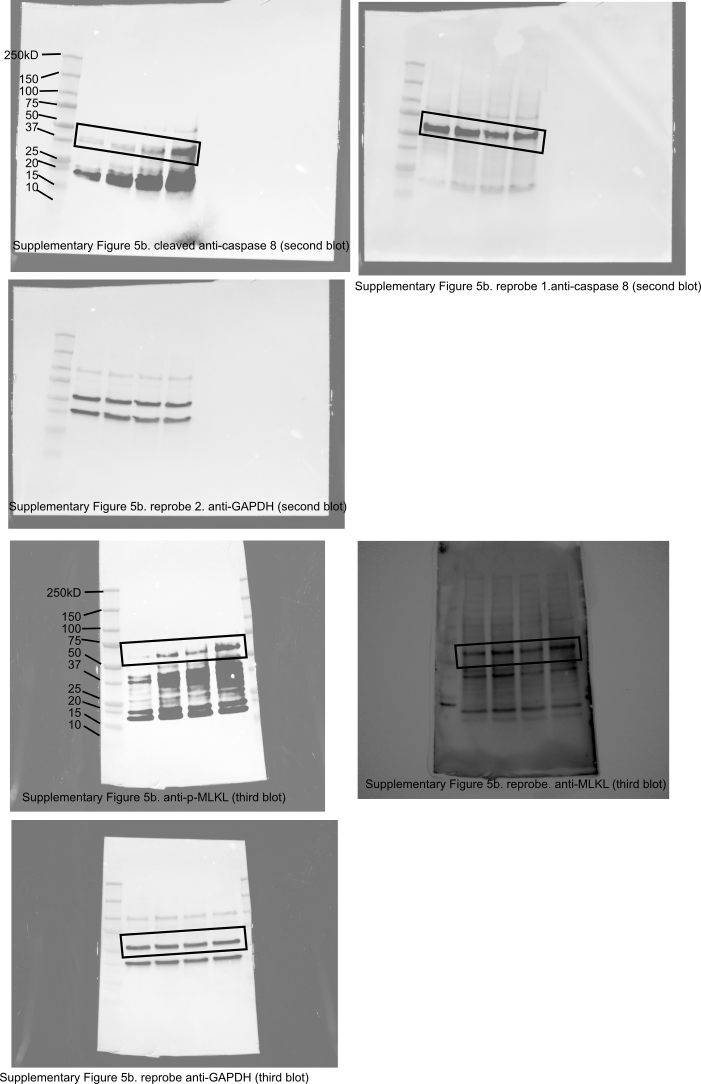


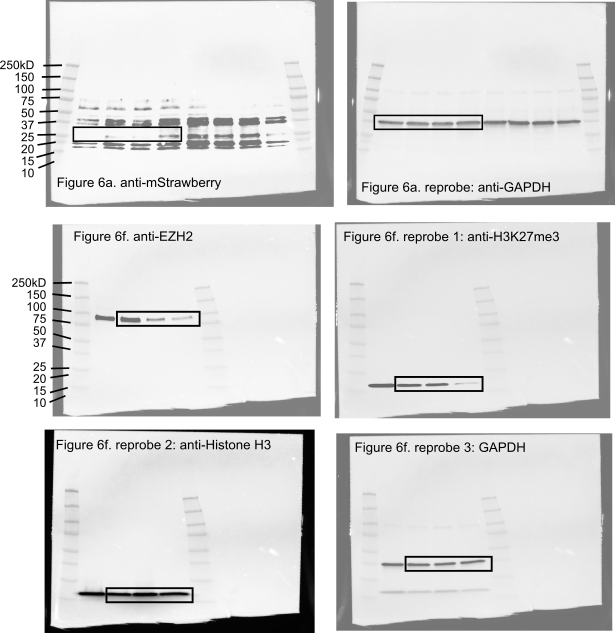


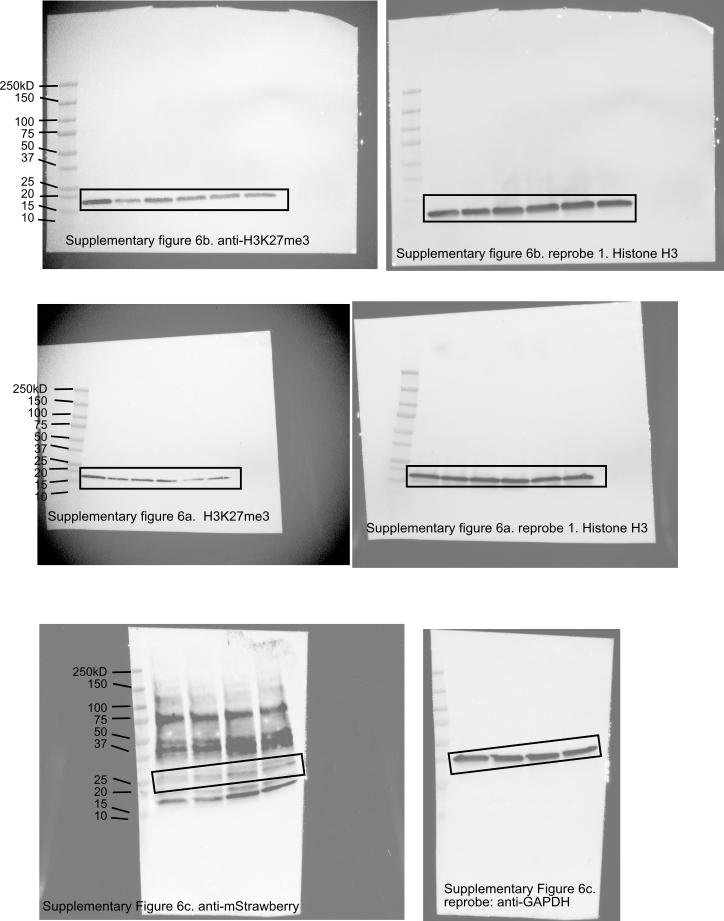


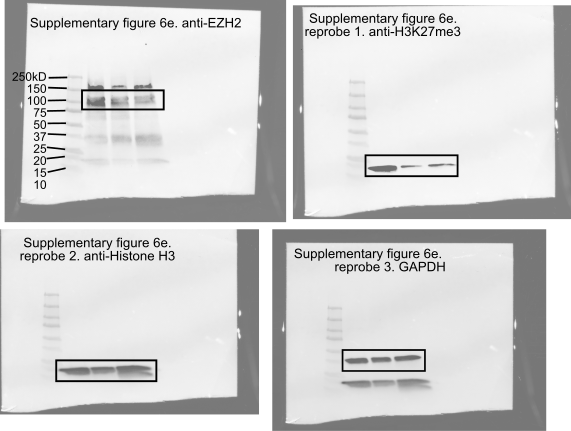

Supplement: Supplementary file 2 — Fig. S7. Full uncropped western blot images. [file MOL2-19-1471-s003.docx]
