# Supplementary material for: Targeting PRAME directly or via EZH2 inhibition overcomes retinoid resistance and represents a novel therapy for keratinocyte carcinoma
Source: Mol Oncol. 2025 Mar 18;19(5):1471–92. doi: 10.1002/1878-0261.13820 (PMC12077289; doi:10.1002/1878-0261.13820)
Supplement: Supplementary file 3 — Table S1. Patient‐derived skin tumor age, diagnosis and sex. Table S2. PRAME shRNA and control shRNA catalogue numbers, sequences and lot numbers. Table S3. PRAME sgRNA and control sgRNA catalogue numbers, target sequences and target region. Table S4. Information regarding PRAME ORF overexpression lentiviral particles and control particles. [file MOL2-19-1471-s001.docx]

**Supplementary Tables**

| **Sample** | **Diagnosis** | **Sex** | **Age** |
| --- | --- | --- | --- |
| 257 | Normal skin | M | 45 |
| 50 | BCC | M | 62 |
| 436-2 | SCC | F | 76 |
| 269 | Actinic keratosis | M | 77 |
| 263 | Bowen’s disease | F | 69 |

**Supplementary Table S1.** **Patient-derived skin tumor age, diagnosis and sex.**

| **Gene Construct** | **Catalogue #** | **Source clone ID** | **Vector** | **Hairpin Sequence** | **Lot #** |
| --- | --- | --- | --- | --- | --- |
| PRAME shRNA #1 | V3SH7590-226107236 | V3SVHS00_6044887 | pSMART hCMV/TurboGFP | CTCTGGGAGTGCTGATGAA | V20102903 |
| PRAME shRNA #2 | V3SH7590-227653485 | V3SVHS00_7591135 | pSMART hCMV/TurboGFP | CTGTTGTAAAGAAACTGTT | V20102903 |
| Non-targeting control (scrambled) | S-005000-01 | V19091301 | hCMV-TurboGFP-NTC | - | V19091301 |

**Supplementary Table S2.** ***PRAME* shRNA and control shRNA catalogue numbers, sequences and lot numbers**.

| **Gene Construct** | **Catalogue #** | **Source Clone ID** | **Vector** | **Genomic location** | **DNA target sequence** | **Lot#** |
| --- | --- | --- | --- | --- | --- | --- |
| Dharmacon Edit-R all-in-one PRAME lentiviral sgRNA | VSGH11937-247607890 | VSGHSOH_28589739_ | pAIOsgRNA hEF1a | Hg38+\|+chr22:22548244-22548266 | GATGTCCTCATAACTCTCCA | V22092301 |
| Dharmacon Edit-R all-in-one lentiviral sgRNA | VSGC11964 | - | pAIOsgRNA hEF1a | - | - | V20081301 |

**Supplementary Table S3.** ***PRAME* sgRNA and control sgRNA catalogue numbers, target sequences and target region.**

| **Gene construct** | **Catalogue #** | **Source clone ID** | **Vector** | **Lot #** | **Gene bank sequence** |
| --- | --- | --- | --- | --- | --- |
| Dharmacon Precision LentiORF PRAME ORF lentiviral particles | OHS5899-202619781 | PLOHS_100006103 | pLOC | V21081102 | DQ893473.2 |
| Dharmacon Precision LentiORF TurboRFP Control lentiviral particles | OHS5833 | - | pLOC | V19060706 | - |

**Supplementary** **Table S4.** **Information regarding PRAME ORF overexpression lentiviral particles and control particles.**

| **Antibody/Target** | **Supplier** | **Catalogue number** | **Dilution** |
| --- | --- | --- | --- |
| PRAME | Abcam | ab219650 | 1:1000 |
| Keratin 1 | Abcam | ab185628 | 1:20000 |
| Keratin 5 | Abcam | ab52635 | 1:10000 |
| Keratin 10 | Abcam | ab76318 | 1:10000 |
| Keratin 13 | Abcam | ab92551 | 1:10000 |
| Keratin 14 | Abcam | ab7800 | 1:1000 |
| Keratin 18 | Abcam | ab133263 | 1:10000 |
| Keratin 19 | Abcam | ab76539 | 1:10000 |
| EZH2 | Abcam | ab150433 | 1:1000 |
| Cleaved Caspase 8 | Cell Signaling Technology | 18C8 | 1:1000 |
| Caspase 8 | Cell Signaling Technology | 4790 | 1:1000 |
| p-MLKL | Cell Signaling Technology | D6H3V | 1:1000 |
| MLKL | Cell Signaling technology | D216N | 1:1000 |
| Cleaved Caspase 9 | Cell Signaling Technology | 52873 | 1:1000 |
| Caspase 9 | Cell Signaling Technology | 9508 | 1:1000 |
| BID | Cell Signaling Technology | 2002 | 1:1000 |
| H3K27me3 | Cell Signaling Technology | C36B11 | 1:1000 |
| Histone 3 | Cell Signaling Technology | D1H2 | 1:2000 |
| Anti-Rabbit IgG HRP-linked antibody | Cell Signaling Technology | 7074 | 1:5000 |
| Anti-Mouse IgG HRP-linked antibody | Cell Signaling Technology | 7076 | 1:5000 |
| mStrawberry | Origene | TA180049 | 1:2000 |
| GAPDH | Thermo Fisher Scientific | PA1-987 | 1:5000 |

**Supplementary** **Table S5.** **Western blotting antibody sources and dilutions.**
